# Synergistic immunochemotherapy targeted SAMD4B-APOA2-PD-L1 axis potentiates antitumor immunity in hepatocellular carcinoma

**DOI:** 10.1038/s41419-024-06699-2

**Published:** 2024-06-17

**Authors:** Feng Qi, Jian Zhang, Jia Li, Donghe Li, Na Gao, Zhuoran Qi, Xiuyan Kong, Zhijie Yu, Yuan Fang, Wenguo Cui, Jinglin Xia

**Affiliations:** 1 National Medical Center & National Clinical Research Center for Interventional Medicine. Liver Cancer Institute, Zhongshan Hospital, Fudan University, 180 Fenglin Road, Shanghai, 200032 China; 2 Department of Oncology, Ruijin Hospital, Shanghai Jiao Tong University School of Medicine, Shanghai, 200025 China; 3 Department of Thoracic Surgery, Ruijin Hospital, Shanghai Jiao Tong University School of Medicine, Shanghai, 20025 China; 4 Shanghai Ji Ai Genetics and IVF Institute, Obstetrics and Gynecology Hospital, Fudan University, Shanghai, 200011 China; 5 Department of Laboratory Medicine, Zhongnan Hospital of Wuhan University, Wuhan University, Wuhan, 430071 China; 6 Zhejiang Key Laboratory of Intelligent Cancer Biomarker Discovery and Translation, First Affiliated Hospital of Wenzhou Medical University, Wenzhou, 325035 China; 7 Department of Liver Surgery, Key Laboratory of Carcinogenesis and Cancer Invasion (Ministry of Education), Liver Cancer Institute, Zhongshan Hospital, Fudan University, Shanghai, 200032 China; 8 Department of Orthopaedics, Shanghai Key Laboratory for Prevention and Treatment of Bone and Joint Diseases, Shanghai Institute of Traumatology and Orthopaedics, Ruijin Hospital, Shanghai Jiao Tong University School of Medicine, Shanghai, 200000 China

**Keywords:** Cancer microenvironment, Targeted therapies

## Abstract

Targeted and immunotherapy combined with interventional therapy can improve the prognosis of advanced cancer patients, and it has become a hot spot to find the new therapeutic schemes, but most of which are not satisfactory. Single-cell RNA sequencing was performed in PDX mouse models with or without TCC therapy. 2-’O-Methylation modification and multiplex immunofluorescence staining were used to explore the function and mechanism of SAMD4B in the immune context of HCC. Here, we propose for the first time a synergistic immunochemotherapy that exerts a potent antitumour effect for patients with advanced hepatocellular carcinoma (HCC) in clinical practice based on three common antitumour drugs and found that HCC patients with new synergistic immunochemotherapy had better three-year overall survival (*p* = 0.004) and significantly higher survival ratio (increased by 2.3 times) than the control group. We further reveal the immunoregulatory mechanism of synergistic immunochemotherapy through 2’-O-Methylation modification mediated by SAMD4B, a tumour suppressor gene. Mechanistically, SAMD4B, increased by the reduced mutations of upstream genes NOTCH1 and NOTCH2, affected the instability of APOA2 mRNA by 2-’O-Methylation modification of the C-terminus. The decreased APOA2 further attenuated programmed death ligand 1 (PD-L1) level with a direct interaction pattern. The high-SAMD4B tumour tissues contained fewer native CD29+CD8+ T cells, which improved immune microenvironment to achieve the effect of antitumour effect. Overall, we developed a potent synergistic immunochemotherapy strategy that exerts an efficient anti-HCC effect inducing SAMD4B-APOA2-PD-L1 axis to inhibit tumour immune evasion.

## Introduction

The combination of targeted and immunotherapy has begun to challenge many types of cancers in the first or second line [1] and achieved amazing results [1–3], especially in advanced tumours [4, 5], chemotherapy intolerance, or resistance to chemotherapy and other situations, providing another high-quality alternative to chemotherapy [2–5]. Such as, the Phase II Single-Arm Clinical CAVE Trial suggested that cetuximab (CET) rechallenge + Avelumab (AVE) for pre-treated patients with RAS wide-type metastatic colorectal cancer (mCRC) was an active and well tolerated rechallenge therapy [6]; the Phase IIa study AVETUXIRI (NCT03608046) demonstrated that the AVE combined with CET and ipilimumab (IPI) for treatment of refractory microsatellite stable (MSS) mCRC had better overall survival rate (OS) and progressive free survival (PFS) [7]; and atezolizumab (ATE) + bevacizumab (BEV) and tremelimumab (TRE) + durvalumab (DUR) have been approved for patients with unresectable hepatocellular carcinoma (HCC), based on the results of IMbrave150 (NCT03434379) [8, 9] and HIMALAYA [10] respectively, both used for first-line therapy. In short, the combination of targeted and immunotherapy is becoming the major trend in the treatment of advanced cancers in the future.

Some studies have shown that the combination of targeted and immunotherapy have synergistic effects [2, 3], and targeted drugs also have significant regulatory effects on tumour immune microenvironment [11, 12]. However, the mechanism of targeted therapy to enhance immunotherapy is still unclear. Therefore, to develop a potential therapeutic strategy to improve antitumour efficacy, it is particularly important to find the specific regulatory network involved. We speculated that TCC cocktail (thalidomide (THA)+carmofur (CAR)+cantharidin (CAN)), also named thalidomide based cocktail (TBC), may be a promising treatment strategy for patients with advanced HCC and it has been preliminarily applied in our clinical treatment successfully. However, there is no clear evidence that these three drugs could be combined to treat patients with advanced HCC. There is few research on this novel clinical combination therapy, especially the mechanism of its anti-HCC effect, either. Therefore, it is necessary to further explore the specific mechanism of this triple drug combination in HCC treatment.

Here, we retrospectively collected the clinical data of 92 patients with unresectable HCC and observed that the efficacy of TCC in clinical practice had better 3-year overall survival (*p* = 0.004) and significantly higher survival ratio (increased by 2.3 times) than the control group. Later, we collected the resected tumours and conducted single-cell RNA sequencing (scRNA-seq). We then validated that SAMD4B, increased by TCC therapy, affected the instability of APOA2 mRNA by 2-’O-Methylation modification, further reducing PD-L1 level with a direct interaction pattern. In addition, the differences of immune cell clusters between low/high SAMD4B in HCC tumours groups revealed the heterogeneity of immune cell composition. We finally verified the above conclusion that TCC therapy could regulate the immune microenvironment by activating SAMD4B to achieve antitumour effects in immunocompetent C57BL6/J mice.

## Results

### TCC therapy showed anti-HCC efficacy in vivo but not in vitro

**Fig. 1:**
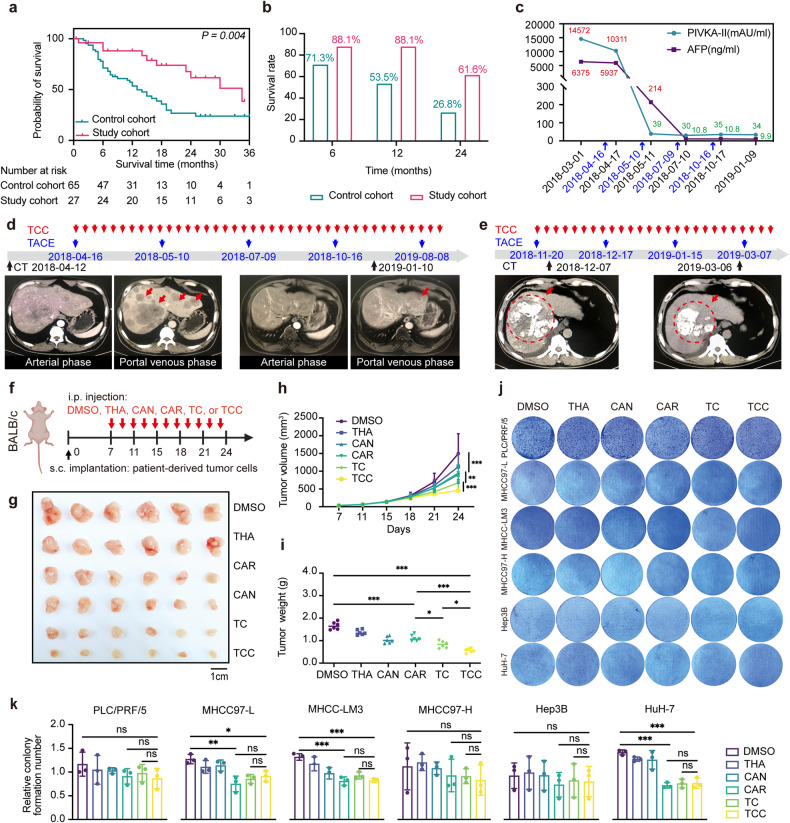
The antitumour effect of TCC treatment in clinical practice andexperimental verification in a mouse PDX model. **a** Kaplan‒Meier survival analysis demonstrated that the study cohort treated with TCC showed better survival. **b** The study cohort demonstrated a better survival ratio at three timepoints. **c** AFP and PIVKA-II expression were significantly decreased in the study cohort. **d** Patient #1 underwent 5 courses of TCC therapy, a significant decrease in tumour number and remarkable tumour volume shrinkage were found by CT scan. **e** Patient #2 underwent 3 courses of TCC therapy, remarkable tumour volume shrinkage was found by CT scan. **f**. **g** Thirty-six PDX models were established by implanting patient-derived tumour cells subcutaneously into the flanks of BALB/C mice. Seven days later, all mice developed tumours of comparable size and were then divided into six groups and injected separately with DMSO, THA, CAR, CAN, TC, or TCC. Comparing tumour size, tumour volume (**h**) and tumour weight (**i**), monotherapy suppressed tumour growth to some extent, but the combination of drugs, especially the TCC combination, further delayed it. **j**, **k** Colony formation assay results. The addition of THA or THA and CAN had no further impact on cell viability in either of the six types of HCC cells. Tukey’s post hoc test (level of significance: ***P < 0.001; **P < 0.01; ns, *P* > 0.05).

To evaluate the efficacy of TCC therapy in clinical practice, we retrospectively collected the clinical data of patients with unresectable HCC diagnosed at Zhongshan Hospital, Fudan University, from May 2017 to May 2020. There were 27 patients in the study group and 65 patients in the control group. All patients were initially treated with transarterial chemoembolization (TACE); additionally, patients in the study group were administered a combined oral medication treatment daily that consisted of THA (50 mg once every night), CAR (100 mg three times a day) and CAN (compound Mylabris capsules 750 mg twice a day), until tumour progression or the development of intolerable side effects. Kaplan‒Meier survival analysis demonstrated that the study group showed better overall survival (Fig. 1a, b). The clinical characteristics of the included patients can be found in Supplementary Data 1. Two patients in the study group showed remarkable tumour volume shrinkage during TCC therapy (Fig. 1c–e).

Next, we explored the potential mechanism of TCC therapy in vivo and in vitro. Thirty-six PDX models were established by implanting patient-derived tumour cells subcutaneously into the flanks of nude mice. Mice were divided into 6 groups and injected separately with DMSO, THA, CAR, CAN, TC (THA+CAR), or TCC (Fig. 1f). As expected, monotherapy suppressed tumour growth to some extent, but the combination of drugs, especially the TCC combination, further delayed tumour growth (Fig. 1g–i).

In colony formation assays, CAR decreased the viability of MHCC97-L, MHCC-LM3, and HuH-7 cells, which was expected because CAR is the precursor of 5-FU, which has been demonstrated to have an6tumour effects [13]. However, the combination of THA and CAR had no further impact on cell viability in any of the six types of HCC cells (Fig. 1j, k). Furthermore, increasing the concentration of THA, CAR, or THA+CAN also had a limited effect on the cell survival rate (Supplementary Fig. 1a–f). All these results indicate that TCC had no effect on mice and humans in vitro.

Overall, the efficacy of TCC therapy was assessed by analysing retrospective cohort data and was confirmed in vivo in mice and humans but not in vitro. The latter discovery might be that the six types of cells tested were not suitable for TCC therapy, or that TCC did not directly kill cells, but regulated the tumour immune microenvironment to produce antitumour effects.

### The pleiotropic changes in response to single-agent and combined therapy suggested that TCC might activate the immune response to tumours

**Fig. 2:**
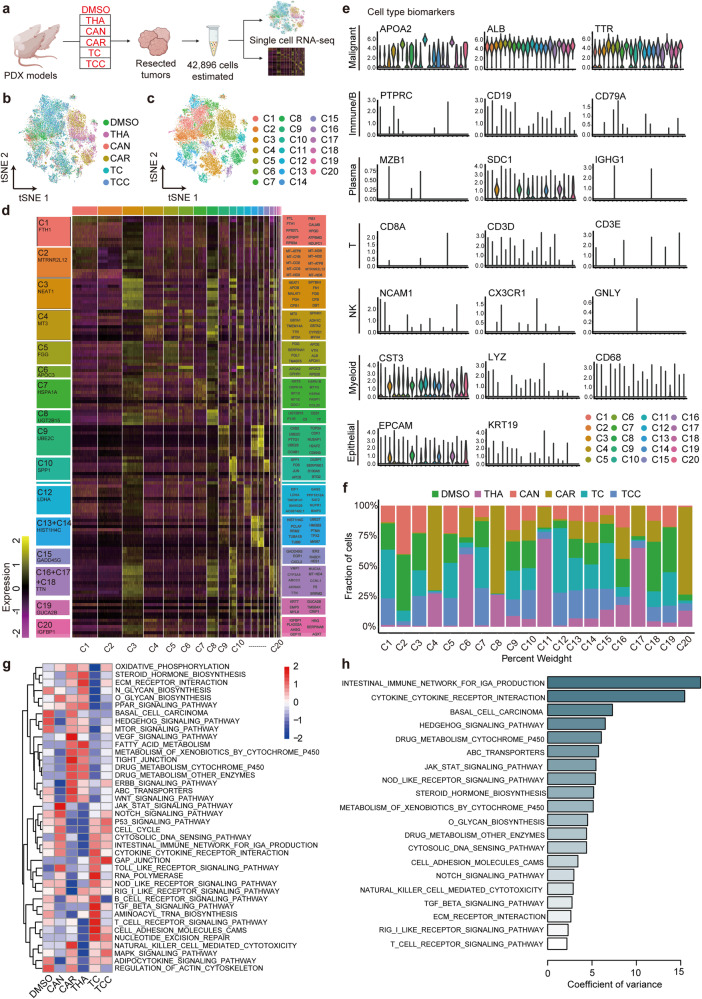
The pleiotropic changes in response to single-agent and combined therapy suggested that TCC might activate the immune response to tumours. **a** We collected resected tumours from mouse PDX models and conducted scRNA-seq. The tSNE plots of DEGs (**b**) and cell clusters (**c**). **d** Twenty main cell clusters were identified among cells from 6 groups with the expression of certain marker genes. **e** These identified cell clusters could be assigned to known cell lineages through 20 marker genes. **f** After observing changes in the number of cells in each cluster after treatment with a single agent or combined agent, different clusters showed pleiotropic changes. Differentially expressed genes among the 6 groups were used to carry out gene enrichment analysis (**g**) and pathway enrichment analysis (**h**).

To explore the association between TCC and the tumour immune microenvironment, we further studied the constructed PDX models (Fig. 1), collected the resected tumours and conducted scRNA-seq. We obtained single-cell transcriptomes for 42,896 cells after quality control (Fig. 2a). Twenty main cell clusters were identified among cells from 6 groups with the expression of certain marker genes (Fig. 2b–d). These identified cell clusters were assigned to known cell lineages based on marker genes: we identified one major cluster as malignant cells as well as clusters of immune cells (B, plasma, T, NK and myeloid cells) and epithelial cells (Fig. 2e, Supplementary Fig. 2a).

After observing changes in the number of cells in each cluster after treatment with single agents or combined agents, we found that different clusters showed pleiotropic changes (Fig. 2f). Some clusters were resistant to a single agent. For example, C4, C6, C8 and C11 were resistant to THA, and cell numbers in those clusters even increased after treatment, but other clusters remained sensitive to THA. This difference in drug response was also observed in both the CAN and CAR monotherapy groups. In the TC group, C2, C16, C17, C18 and C20 were sensitive to TC, while C1, C4, C5, C7, C12, C13 and C15 demonstrated an increase in cell numbers after treatment. In the TCC group, C2, C9, C16, C18 and C19 showed a decrease in cell numbers after treatment, while C1, C4, C6, C7, C11, C12, C13, C14, C17 and C20 exhibited significant resistance to treatment (Fig. 2f). Therefore, we confirmed that the actual effect of drugs on tumour cells in vivo was more complex, so their effectiveness could not be verified only through in vitro experiments.

Gene enrichment analysis based on the differentially expressed genes among the 6 groups was carried out; notably, in the TCC group, tumour-related signalling pathways were upregulated, including the ErbB, Notch, p53, and MAPK signalling pathways, cell cycle, nucleotide excision repair, and cell adhesion molecules (CAMs) (Fig. 2g). The cell cycle patterns of all the clusters were further analysed, and most of the cells were in the G0/G1 phase (Supplementary Fig. 2b, c). However, the cell cycle patterns of C16 and C18 were different from those of the other clusters, with a higher proportion of G2/M and S phase cells (Supplementary Fig. 2d, e).

In addition to tumour-related signalling pathways, immune-related signalling pathways were also upregulated in the TCC group, including the cytokine DNA sensing pathway, intestinal immune network for IgA production, NOD-like receptor signalling pathway, TGF-beta signalling pathway, and natural killer cell-mediated cytotoxicity (Fig. 2g). The top two enriched signatures, intestinal immune network for IgA production and cytokine‒cytokine receptor interaction, were also immune-related signatures (Fig. 2h).

In the tumours resected from the PDX models, in addition to human-derived cells, we also obtained some murine-derived cells, which were represented by nine cell clusters (Supplementary Fig. 3a, b). These identified cell clusters were assigned to known cell lineages based on marker genes: we identified major clusters of fibroblasts and macrophages as well as clusters of innate lymphoid cells (ILCs), endothelial cells, stromal cells and monocytes (Supplementary Fig. 3c, d). Moreover, all of these identified murine-derived cells were mainly in the G0/G1 phase (Supplementary Fig. 3e–g). Although the immune function of nude mice was deficient, we tentatively concluded that TCC activated the immune response to tumours in nude mice.

Overall, the cell clusters identified by scRNA-seq showed pleiotropic changes in response to single-agent and combination therapy, indicating that TCC might activate the immune response to tumours.

### The C16+C18 cluster was the TCC-targeted cell cluster

**Fig. 3:**
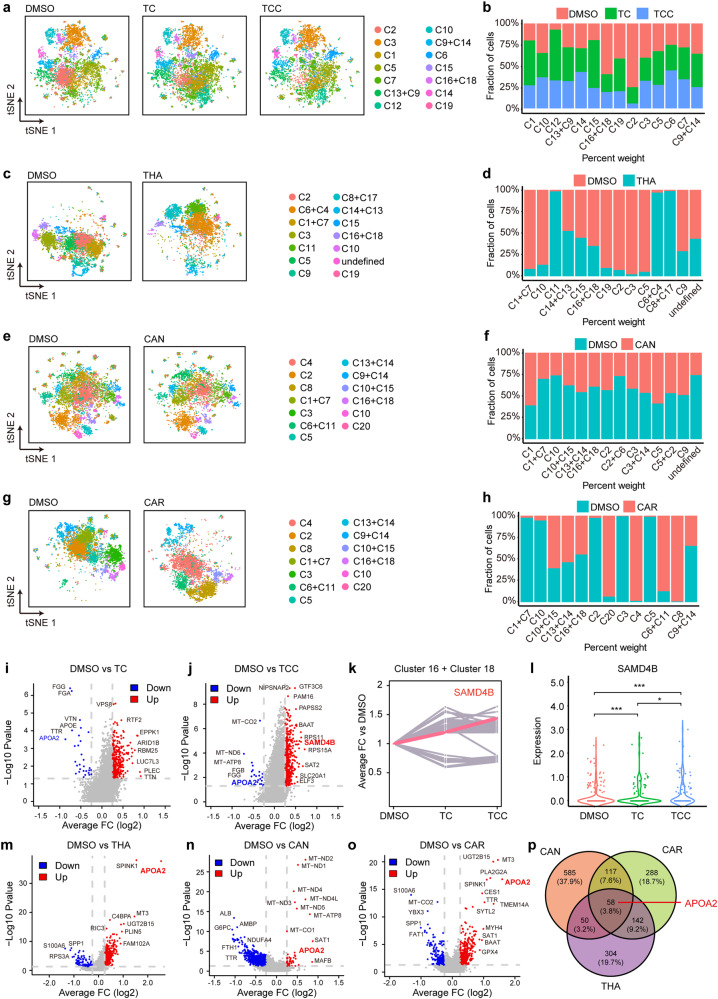
HCC patients with the C16+C18 cluster might be the target population of our novel triple-drug therapy. **a**, **c**, **e**, **g** The tSNE plots of the DMSO group compared with the single-agent and combined-agent groups separately. **b**, **d**, **f**, **h** The fraction of cells in the DMSO group compared with the single-agent and combined-agent groups separately. Only the C16+C18 cluster was the main target cell cluster of TCC. SAMD4B was the most significantly differentially expressed gene in DMSO vs. TC (**i**) and DMSO vs. TCC (**j**). **k**, **l** The expression of SAMD4B in the DMSO, TC and TCC groups gradually increased. Differentially expressed gene analysis of DMSO vs. THA (**m**), DMSO vs. CAN (**n**), and DMSO vs. CAR (**o**). **p** There were 58 differentially expressed genes shared by the three compared groups, and APOA2 was the most significant gene.

To further determine the role of TCC in the immune response towards tumours in humans, we compared the DMSO group with the single-agent and combined-agent groups separately (Fig. 3a–h, Supplementary Fig. 4). Two clusters, C2 and C16+C18, were found to be sensitive to the combined therapy, showing a gradual decrease in the number of cells in the DMSO, TC and TCC groups (Fig. 3b). THA showed significant efficacy in the C1+C7, C10, C19, C2, C3, and C5 clusters (Fig. 3d); CAN showed efficacy in all identified cell clusters (Fig. 3f); and CAR demonstrated remarkable efficacy in the C20, C4, C6+C11, and C8 clusters (Fig. 3h). According to the above results, we found that only the C16+C18 cluster was the main target cell cluster of TCC; that is, this cluster was sensitive to combined therapy but had no significant response to the three monotherapies. This finding was consistent with the tumour regression phenomenon observed in our PDX models (Fig. 1g–i). Therefore, we suggest that HCC patients with the C16+C18 cluster might be the target population of our novel triple-drug therapy.

In the gene enrichment analysis of the combined group, we found an enrichment of immune-related pathways, including the JAK/STAT signalling pathway, NOD-like receptor signalling pathway, T-cell receptor signalling pathway, TGF-beta signalling pathway, cytokine‒cytokine receptor interaction, and Toll-like receptor signalling pathway, as well as tumour-related pathways, including the CAM, Notch, MAPK, and ErbB signalling pathways (Supplementary Fig. 5a, b). This further confirmed our hypothesis that the inhibitory effect of TCC on tumours might be related to the tumour immune microenvironment. Drug metabolism cytochrome P450 (CYP) was enriched in all three single-agent groups (Supplementary Fig. 5c–h). The overexpression of CYP in tumour cells has been shown to accelerate the metabolism of antitumour drugs, which might lead to drug resistance and be related to recurrence and poor prognosis [14]. Therefore, we speculated that the inhibitory effect of single-agent therapy on tumours was not obvious due to the upregulation of CYP in tumour cells.

To investigate the mechanism of action of TCC on C16+C18 cells, we analysed differentially expressed genes in the combined therapy group (Fig. 3i, j). SAMD4B was the most significantly differentially expressed gene. More importantly, its expression level in the DMSO, TC and TCC groups gradually increased, consistent with the therapeutic trend in the PDX model (Fig. 3k, l). In addition, we investigated the reason why C16+C18 cells were resistant to single-agent therapy and found that the three groups shared 58 differentially expressed genes (Fig. 3m–o), among which APOA2 was the most significant gene (Fig. 3p).

### SAMD4B acted as a tumour suppressor by targeting the APOA2 oncogene

**Fig. 4:**
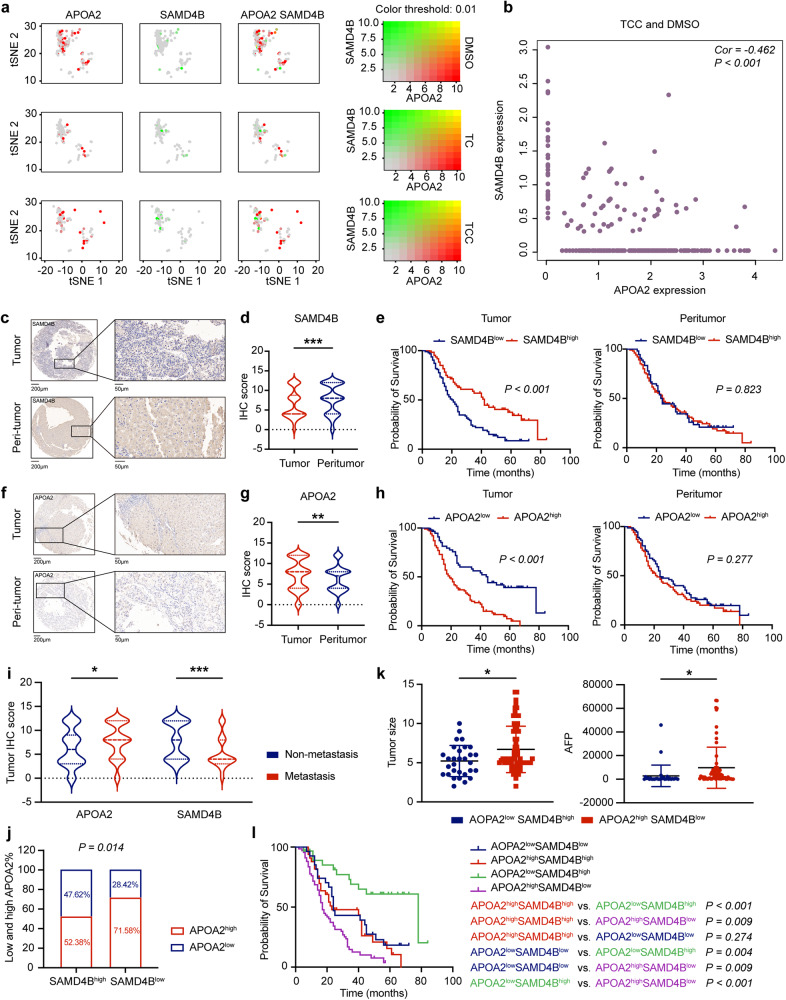
SAMD4B acted as a tumour suppressor by targeting the APOA2 oncogene. **a** The tSNE plots of APOA2 and SAMD4B in the DMSO, TC and TCC groups. With the gradual increase in the number of combined drugs, the expression level of APOA2 gradually decreased, while that of SAMD4B gradually increased. **b** There was a significant negative correlation between them. **c**, **d** SAMD4B was highly expressed in the adjacent tissues in 158 HCC patients, **e** and the patients with high SAMD4B expression in tissues had a better prognosis than those with high expression in adjacent tissues. **f**, **g** APOA2 was significantly expressed at low levels in adjacent tissues, **h** and patients with low APOA3 expression only in cancer tissues had a better prognosis. **i** Patients with high APOA2 were more likely to exhibit metastasis, while patients with high SAMD4B were less likely to exhibit metastasis. **j** High-SAMD4B-expression patients were more likely to have low APOA2 expression. **k** Patients with high APOA2 and low SAMD4B expression had larger tumours and AFP. **l** The group with high APOA2 and low SAMD4B expression had the worst prognosis, while the group with low APOA2 and high SAMD4B expression had the best prognosis. Student’s *t* test (level of significance: ***P < 0.001; **P < 0.01; *P < 0.05).

To explore the association between SAMD4B and APOA2, we assessed their expression levels using scRNA-seq. With the gradual increase in the DMSO, TC and TCC groups, the expression level of APOA2 gradually decreased, while that of SAMD4B gradually increased (Fig. 4a), and there was a significant negative correlation between them (Fig. 4b).

Then, we retrospectively explored the expression levels of SAMD4B and APOA2 in an HCC cohort. The cancer and adjacent tissues of 158 patients with HCC were collected, and immunohistochemical staining assays were performed (Supplementary Fig. 6). We found that SAMD4B was highly expressed in adjacent tissues (Fig. 4c, d) and that the patients with high SAMD4B expression in cancer tissues had a better prognosis than those with low SAMD4B expression in adjacent tissues (Fig. 4e). However, APOA2 was significantly expressed at low levels in adjacent tissues (Fig. 4f, g), and only patients with low APOA2 expression in cancer tissues had a better prognosis (Fig. 4h). The above results suggested that SAMD4B might be a tumour suppressor gene, while APOA2 might be an oncogene.

We also checked the correlation between the two genes and clinical characteristics. Patients with high APOA2 expression levels were more likely to exhibit metastasis, while patients with high SAMD4B were less likely to exhibit metastasis (Fig. 4i). There was a significant correlation between the two genes in HCC patients; that is, patients with high SAMD4B expression were more likely to have low APOA2 expression (Fig. 4j). In addition, patients with high APOA2 and low SAMD4B expression levels had larger tumours and higher AFP levels (Fig. 4k). It is well known that both tumour size and AFP level are HCC biomarkers related to prognosis. Similarly, the group with high APOA2 and low SAMD4B expression levels had the worst prognosis, while the group with low APOA2 and high SAMD4B expression levels had the best prognosis (Fig. 4l). Therefore, we speculated that the reason for the good efficacy of TCC might be that it could activate SAMD4B and inhibit APOA2.

### 2’-O-Methylation modification of APOA2 mRNA by SAMD4B

Next, we further verified the interaction mechanism between SAMD4B and APOA2. We transfected SAMD4B and APOA2 plasmids into HEK293T cells and observed the mRNA instability and expression of APOA2 by overexpression and knockout of SAMD4B, respectively.

**Fig. 5:**
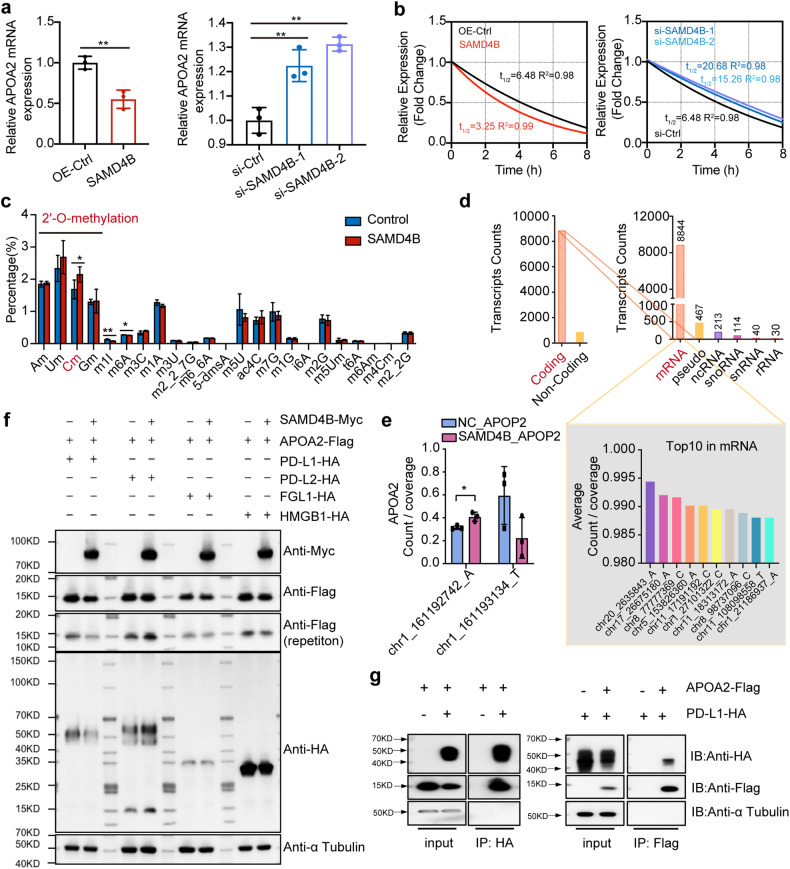
2’-O-methylation of APOA2 mRNA by SAMD4B. The mRNA instability (**a**) and expression (**b**) of APOA2 were observed by overexpression and knockout of SAMD4B, respectively. The overexpression of SAMD4B increased the instability of APOA2 mRNA and downregulated the expression of APOA2, while SAMD4B knockdown reduced the instability of APOA2 mRNA and activated APOA2. **c** All known types of RNA modifications were detected by liquid chromatography‒mass spectrometry, and three modifications, namely, 2’-O-methylation, m1I and m6A, showed significant differences. **d** The 2’-O-methylation modification in the treatment group mainly occurred in the coding region, that is, the mRNA region. **e** Two modification sites located in APOA2 changed based on a comparison of the control and treatment groups. **f** SAMD4B was changed to check the expression of downstream APOA2 and four immune checkpoints. Only PD-L1 was consistent with APOA2, and the others were unchanged. **g** A coimmunoprecipitation (Co-IP) method was used to verify the direct interaction between APOA2 and PD-L1 from both positive and negative aspects. Student’s *t* test (level of significance: **P < 0.01; *P < 0.05).

The overexpression of SAMD4B downregulated the expression of APOA2; when SAMD4B was knocked out, upregulated APOA2 expression was observed (Fig. 5a). The results showed that they did have a negative regulatory relationship at the transcriptome level. Furthermore, the overexpression of SAMD4B increased the instability of APOA2 mRNA, which was reduced by SAMD4B knockdown (Fig. 5b). This was consistent with previously reported research showing that SAMD4B can regulate the instability of mRNA [15], but the research on whether it is an RNA-modifying enzyme is unclear.

RNA methylation is a typical epigenetic modification through which gene stability is modulated. We transfected plasmids carrying the SAMD4B and APOA2 genes into HEK293T cells. The control group overexpressed APOA2, and the treatment group overexpressed both SAMD4B and APOA2. All known types of RNA modifications were assessed by liquid chromatography‒mass spectrometry, and three modifications, namely, 2’-O-Methylation, m1I and m6A, were shown to have significant differences (Fig. 5c). Among them, only the results for 2’-O-Methylation were consistent with our expectations, and the modification of the C-terminus was significantly increased after transfection with a plasmid overexpression SAMD4B. Therefore, we hypothesized that SAMD4B regulated APOA2 through 2’-o-methylation of the C-terminus.

Then, we sequenced the 2’-O-Methylation modification in the treatment group and found that the modification mainly occurred in the coding region, that is, the mRNA region (Fig. 5d). The sites chr20_2635843_A, chr17_26675180_A, chr8_77777369_C, chr5_153826360_A, chr11_17191192_C, chr1_27101322_C, chr11_18313172_A, chr8_98737096_C, chr11_108098558_T and chr1_21186937_A were the top 10 sites with the highest average 2’-O-methylation modification values. Two modification sites located in APOA2 changed between the control and treatment groups (Fig. 5e). The locus chr1_ 161192742_ A was located in the exon region of APOA2 and was significantly changed in the treatment group, so this locus was considered to be the site where SAMD4B carried out 2’-O-Methylation of APOA2, thus affecting the instability of APOA2 mRNA and reducing its abundance.

We suggest that TCC can inhibit the expression of the oncogene APOA2 by activating the tumour suppressor SAMD4B. APOA2 has been reported to potentially be related to immune escape or related to immune checkpoints [16]. Moreover, in Fig. 2, even based on the murine-derived cells, it was clear that the related immune cells had changed after TCC treatment. We simultaneously ectopically expressed Myc-tagged SAMD4B, Flag-tagged APOA2 and HA-tagged immune checkpoints that are mainly expressed in tumours (PD-L1, PD-L2, FGL1 or HMGB1) and demonstrated that overexpression of SAMD4B could reduce the protein level of APOA2. Among the relevant immune checkpoints, especially those expressed in tumours, only PD-L1 was affected by SAMD4B and APOA2, and its expression was positively correlated with that of APOA2 (Fig. 5f). Therefore, we assumed that SAMD4B further affected PD-L1 by regulating APOA2 to achieve immune regulation. To verify whether APOA2 directly regulates PD-L1, we performed a coimmunoprecipitation (co-IP) assay in HEK293T cells to explore whether APOA2 physically binds to PD-L1 and found that the 2 proteins interacted with each other directly. These results suggested that APOA2 regulated PD-L1 through direct interaction (Fig. 5g).

In addition, we explored the potential upstream regulatory mechanism of SAMD4B by analysing our previous cohort, ZS-SEQ-HCC, which consisted of 30 HCC patients. We carried out transcriptomics and whole-exome capture sequencing and divided the cohort into high- and low-SAMD4B-expression groups (Supplementary Fig. 7a); we then ranked genes from high to low according to their mutation frequencies. Supplementary Fig. 7b shows the top 12 genes with the highest mutation frequency, of which NOTCH2 was the gene with the most mutations. These mutations only occurred at the DNA level, and whether they could affect the downstream transcriptome level and even the protein level was unknown. We next observed the expression of genes with high mutation frequency. Only NOTCH2 and NOTCH1 were significantly expressed in the low- and high-SAMD4B-expression groups (Supplementary Fig. 7c) and were negatively correlated with SAMD4B expression (Supplementary Fig. 7d-e). A recent study reported that mutations in NOTCH1 and NOTCH2 are associated with immune escape [17]. The tumour mutational burden (TMB) in the 30 ZS-SEQ-HCC cohort patients was determined because of increased interest in the association between TMB and immunotherapy response. A high TMB was found to be significantly related to the response of various tumours to immunotherapy. For whole-exome capture sequencing, the TMB value was directly related to the number of nonsynonymous mutations, and the TMB level was also related to the number of new tumour antigens. The low-SAMD4B-expression group exhibited a rather high TMB (Supplementary Fig. 7f). Cell type enrichment analysis was performed using gene expression data, and CD8 T cells were significantly enriched in the low-SAMD4B-expression group (Supplementary Fig. 7g). In addition, the level of SAMD4B expression affected the levels of immune checkpoints, such as CD274 and PDCD1LG2 (Supplementary Fig. 7h). All the results indicated that SAMD4B expression affected the mutation of upstream oncogenes NOTCH1 and NOTCH2 and the immune microenvironment. NOTCH might be the upstream regulatory gene of SAMD4B.

### High SAMD4B expression reduced PD-L1 and thus weakened the immune escape of tumour cells from naive CD29+CD8+ T cells

To understand the details of its immune regulation mechanism, such as which immune checkpoints such as PD1 (PD-L1 receptor regulated by SAMD4B), we selected four patients from the low- and high-SAMD4B-expression groups for scRNA-seq (Supplementary Fig. 8). In addition to epithelial/cancer cells, immune cells, including myeloid-derived cells, T cells, B cells, stromal cells, and natural killer (NK) cells, were identified (Supplementary Fig. 9a). All these cell subtypes were shared among patients and between the low- and high-SAMD4B-expression groups, albeit at different proportions (Supplementary Fig. 9b, c). The immune cell clusters varied among patients and low/high-SAMD4B-expression groups, revealing the heterogeneity of immune cell compositions among patients with HCC tumours.

**Fig. 6:**
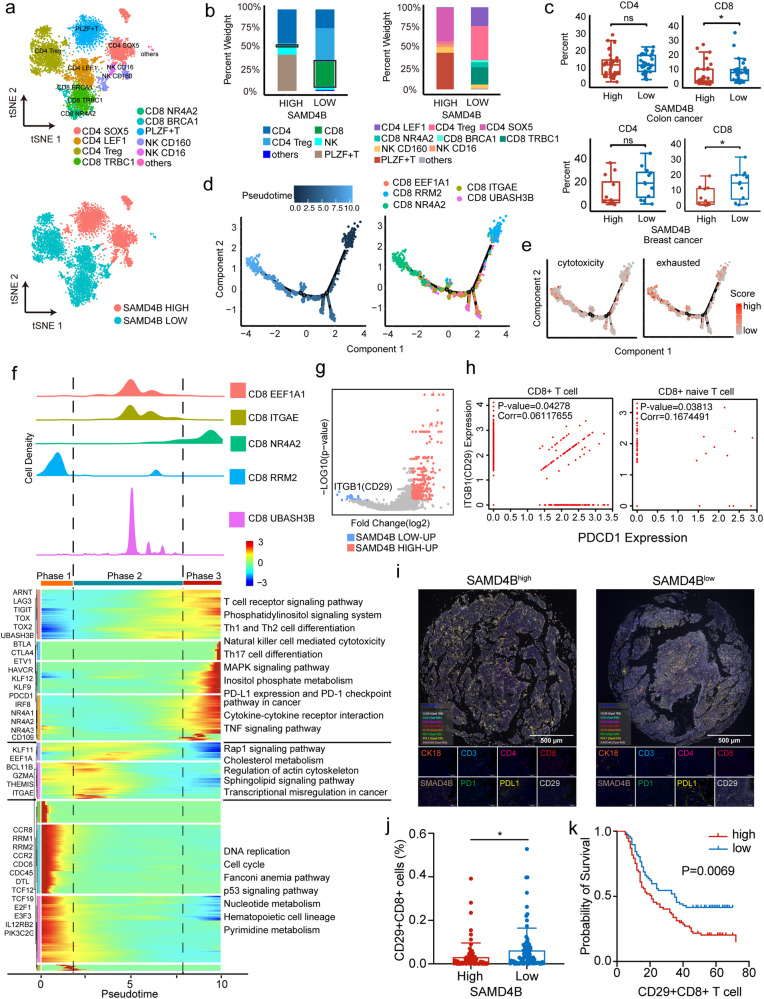
The expression of SAMD4b influenced PD-L1 and thus the immune escape of tumour cells from naive CD29+CD8+ T cells. **a** The tSNE plot of unsupervised clustering of T cells and NK cells. **b** CD8+ T cells (CD8 TRBC1, CD8 NR4A1, CD8 NR4A2 and CD8BRCA1) were enriched in tumour tissues of the low-SAMD4B-expression group. **c** CD8 T cells were significantly enriched in the low-SAMD4B-expression group in breast cancer and colon cancer samples. **d** The pseudotime analysis showed that CD8 RMP2 cells occurred at the beginning of the trajectory path, whereas CD8 NR4A2 cells were found at a terminal state. **e** The exhausted signature was upregulated, whereas the cytotoxic signature had no obvious down- or upregulation trend. **f** The transcriptional changes associated with transitional states were investigated, and the CD8+ T-cell clusters could be categorized into 3 phases. **g** DEG analysis showed that ITGB1 (CD29) was the top upregulated gene in CD8+ T cells in the low-SAMD4B-expression group. **h** In CD8+ and CD8+ naive T cells, the expression of ITGB1 (CD29) showed a significant positive correlation with PDCD1. **i** PD-L1, CK18 and SAMD4B had a colocalization relationship, and PD1 was colocalized with CD29, CD3 and CD8. The low-SAMD4B-expression samples contained more CD29+CD8+ T cells (**j**) in the HCC retrospective cohort, and the high expression of CD29+CD8+ T cells was associated with a worse prognosis (**k**). Student’s *t* test (level of significance: **, *P* < 0.01; *, *P* < 0.05; ns, *P* > 0.05).

We next performed unsupervised clustering of T cells and NK cells. The reclustering revealed 11 populations of cells, including two NK subtypes (NK CD16 and CD160), three subtypes of CD4+ T cells (CD4 SOX5, CD4 LEF1 and Treg), three clusters of CD8+ T cells (CD8 TRBC1, CD8 NR4A1, CD8 NR4A2 and CD8BRCA1), PLZF + T cells and other cells (Fig. 6a and Supplementary Fig. 9d). All these subtypes were shared across patients and between the low- and high-SAMD4B-expression groups. However, CD8+ T cells were enriched in tumour tissues of the low-SAMD4B-expression group (Fig. 6b). Similarly, we found consistent results in breast cancer and colon cancer datasets, in which CD8+ T cells were significantly enriched in the low-SAMD4B-expression group (Fig. 6c and Supplementary Fig. 9e, f). In contrast, the proportions of CD4+ T cells displayed no difference (Fig. 6b). The ratio of CD4/CD8 cells is related to immunogenicity [18]. Generally, the higher the ratio is, the stronger the immunogenicity [19]. Therefore, the high-SAMD4B-expression group contained fewer CD8+ T cells, with a higher ratio and stronger immunogenicity. We next explored the dynamic immune states and cell transitions in HCC-infiltrated CD8+ T cells by inferring the state trajectories using Monocle. Pseudotime analysis showed that CD8+ RMP2 cells occurred at the beginning of the trajectory path, whereas the CD8+ NR4A2 cells were in a terminal state (Fig. 6d). This transition was determined to initiate with CD8+ RMP2 cells through an intermediate cytotoxic state characterized by CD8+ EEF1A1, CD8+ ITGAE and CD8+ UBASH3B cells and finally reach an exhausted state characterized by CD8+ NR4A2 cells. We confirmed that the exhausted signature was upregulated, whereas the cytotoxic signature had no obvious down- or upregulation trend (Fig. 6e).

We then investigated the transcriptional changes associated with transitional states and observed that the CD8+ T-cell clusters could be categorized into 3 phases. CD8 RRM2 cells were predominantly phase 1 cell, characterized by upregulated expression of CCR8, RRM1, RRM2, and CDC45 and low expression of TIGIT, TOX, and TOX2, suggesting that these cells had the lowest cytotoxic capacities (Fig. 6f). Pathway analysis indicated that the p53 signalling pathway was enriched in phase 1. Phase 2 was characterized by increased expression levels of classical cytotoxic genes. Pathway analysis suggested that cholesterol metabolism increased, which played an important role in the activation, clone proliferation and effector function of CD8+ T cells, especially in their antitumour effect [20]. Phase 3 was characterized by high levels of T-cell exhaustion-related TFs, including TOX, TOX2, ARNT, and ETV1, and genes involved in the PD-1 and cytotoxicity pathways, further confirming the exhausted state of these cells. Interestingly, the checkpoint molecule PDCD1 was only modestly upregulated in exhausted states (phase 3). CD8+ T cells from the low-SAMD4B-expression group were primarily characterized as phase 1 and phase 2, with only a few representing the exhausted phase 3, indicating a resident-memory phenotype. In contrast, CD8+ T cells in the high-SAMD4B-expression group were predominantly characterized as phase 3, representing the transition process from cytotoxic to exhausted states (Supplementary Fig. 10a). We concluded that although the low-SAMD4B-expression group had more CD8+ T cells (Fig. 6c), most of them were naive, so they could not play a role in immune regulation.

To further investigate the unique transcriptional states of CD8+ T cells in the low-/high-SAMD4B-expression groups, we determined the expression scores of costimulatory, exhausted and tissue-resident T-cell phenotypes. Costimulatory genes, such as CD226, TNFRSF14, TNFRSF9, and CD69, were increased in high-SAMD4B-expression patients, whereas exhausted and tissue-resident molecules, such as CTLA4 and RUNX3, were reduced in low-SAMD4B-expression patients (Supplementary Fig. 9g). Differentially expressed gene (DEG) analysis revealed that ITGB1 (CD29) was the top upregulated gene in CD8+ T cells in the low-SAMD4B-expression group (Fig. 6g), which might be related to the PPAR signalling pathway being significantly reduced in the low-SAMD4B-expression group (Supplementary Fig. 10b). In CD8+ and CD8+ naive T cells, the expression of ITGB1 (CD29) showed a significant positive correlation with PDCD1 (Fig. 6h), suggesting that CD29+CD8+ T cells might participate in this immune regulation pathway.

To verify this conjecture, we carried out polychromatic immunofluorescence on 8 markers, including cancer cells (CK18), T cells (CD3, CD4, CD8 and CD 29), SAMD4B, PD1 and PD-L1. Figure 6i demonstrates that PD-L1, CK18 and SAMD4B had a colocalization relationship, and PD1 colocalized with CD29, CD3 and CD8. Through the analysis of a tissue microarray of a retrospective cohort (158 HCC patients), the low-SAMD4B-expression samples contained more CD29+CD8+ T cells (Fig. 6j), and the high expression of CD29+CD8+ T cells had a worse prognosis (Fig. 6k). In addition, the low-SAMD4B-expression samples also had more CD3+CD8+ T cells and a higher percentage of PD-L1+CK18+ malignant cells (Supplementary Fig. 10c), which led to a worse prognosis (Supplementary Fig. 10d).

In conclusion, SAMD4B’s regulation of the immune microenvironment was mainly reflected in the fact that when its expression was low, PD-L1 increased, and naive CD29+CD8+ T cells increased. However, high SAMD4B expression could increase the instability of APOA2 mRNA, further reducing PD-L1, and thus weakening the immune escape of tumour cells from naive CD29+CD8+ T cells.

### The tri-drug combination exhibited potent antitumour capacity in immunocompetent mice

**Fig. 7:**
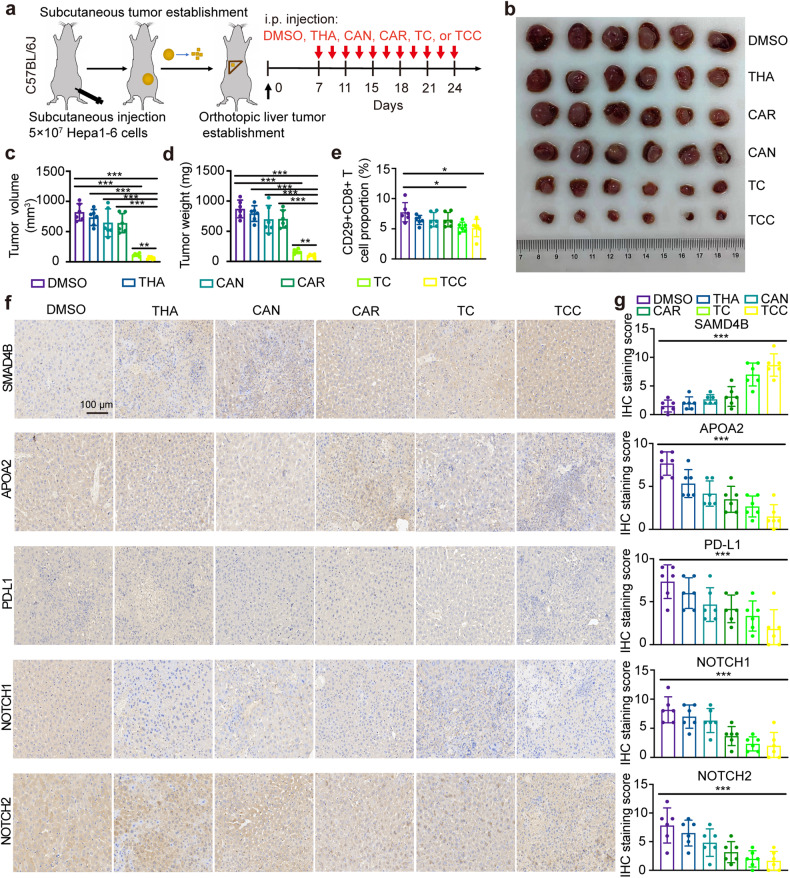
The efficacy and mechanism of TCC treatment were verified in immunocompetent mice. **a** Orthotopic tumour model and drug treatment in C57BL6/J mice. **b** Compared with the single drugs and the dual-drug combination, the tri-drug combination resulted in smaller tumours, regardless of tumour volume (**c**) and weight (**d**). **e** The flow cytometry analysis demonstrated that the lowest proportion of CD29+CD8+ T cells was associated with the tri-drug combination. **f**, **g** Combined treatment with increasing doses of drugs upregulated SAMD4B expression and downregulated the expression of APOA2, PD-L1, NOTCH1 and NOTCH2. Student’s *t* test (level of significance: ***P < 0.001; **P < 0.01; *P < 0.05).

We finally verified the above conclusion that the triple-drug therapy could regulate the immune microenvironment by activating SAMD4B to achieve antitumour effects in immunocompetent C57BL6/J mice. Briefly, 5 × 10^7^ Hepa1-6 cells were injected subcutaneously into C57BL6/J mice. After 3 weeks, the mice were euthanized, and tumours were excised and diced and then orthotopically transplanted into the livers of recipient mice. Seven days later, mice were injected separately with DMSO, THA, CAR, CAN, TC, or TCC (Fig. 7a). Compared with the single drugs and the dual-drug combination, the tri-drug combination resulted in smaller tumours (Fig. 7b), regardless of tumour volume and weight (Fig. 7c, d), which further indicated that TCC had the strongest tumour inhibition effect. Subsequently, flow cytometry analysis demonstrated that the lowest proportion of CD29+CD8+ T cells was associated with the triple drug combination, and the proportion gradually decreased with increasing combined drugs (Fig. 7e and Supplementary Fig. 11). We further verified that SAMD4B could be activated by TCC therapy to achieve an antitumour effect, which was knocked down or overexpressed in immunocompetent C57BL6/J mice (Supplementary Fig. 12a–d). We also examined the expression of genes involved in the regulatory pathway. The combination treatment upregulated SAMD4B expression, downregulated the expression of APOA2, PD-L1, NOTCH1 and NOTCH2 (Fig. 7f, g) and improved the effect of single-drug treatments, consistent with our previous results.

## Discussion

For the first time, we proposed that TCC treatment is a promising treatment strategy for patients with advanced HCC. It is well known that THA inhibits the activity of basic fibroblast growth factor-2 (bFGF), thereby inhibiting angiogenesis [21, 22]. THA also has other antitumour properties and has been established as the standard of care in the treatment of myeloma [23, 24]. In practice, clinicians have attempted to use THA to treat patients with advanced HCC [25]. THA combined with tegafur or uracil has been reported to have moderate effects in the treatment of patients with advanced HCC [26]. In recent years, metronomic chemotherapy has been gradually recognized [27]. It not only disrupts the process of cell division, which inhibits the proliferation of cancer cells, but also eliminates endothelial cells involved in angiogenesis, termed an anti-angiogenetic effect [28]. 5-Fluorouracil (5-FU) with the metronomic pattern could be slowly released in the body from CAR, conferring a marked anti-angiogenetic effect [13, 27]. CAN has been used as a traditional Chinese medicine for >2000 years [29]. Some studies have reported that there are several methods to reduce the side effects of CAN, thus greatly improving its clinical application [30, 31]. Later, CAN was found to reduce the toxicity of other antitumour drugs [32]. In a recent study, CAN was shown to possess antitumour effects against HCC cells both in vivo and in vitro [29]. Although the three drugs have proven their value in antitumour therapy, the strategy of combining the three drugs has not been reported thus far, let alone the mechanism of its anti-hepatocellular effect. Therefore, we proposed for the first time that TCC strategy is a promising treatment strategy for patients with advanced HCC and has been successfully applied to our clinical therapeutics.

SAMD4B is a mammalian homologue of the Smaug protein of *Drosophila melanogaster* and can participate in the process of regulating RNA stability as a posttranscriptional inhibitor [15]. SAMD4B is expressed in most human embryos and adult tissues [15]. Although SAMD4B has been proven to affect the stability of posttranscriptional RNA, its specific mechanism of action has not been reported. We first identified the HCC cell subgroup C16+C18 cell cluster that was sensitive to TCC therapy and determined that the expression level of the SAMD4B gene significantly increased and the expression level of APOA2 significantly decreased. We not only verified that the expression of SAMD4B was negatively correlated with APOA2 but also further verified that SAMD4B affected the expression of APOA2 through 2’-O-Methylation modification. Furthermore, we found that SAMD4B, especially its expression level, could affect the immune microenvironment. The CD4/CD8 ratio is related to immunogenicity. The higher the proportion is, the stronger the immunogenicity. Therefore, the high-SAMD4B-expression group had fewer CD8 cells, a higher ratio and stronger immunogenicity. Then, we used Monocle to infer the state trajectory, discussed the dynamic immune state and cell transformation of CD8+ T cells infiltrated by HCC, and studied the transcriptional changes related to the transition state. We could draw the following conclusion: although there were more CD8+ T cells in the low-SAMD4B-expression group, most of the cells were in an immature state, and they could not play the role of immune regulation.

In this study, we found that after using TCC to treat patients with advanced HCC, the expression of SAMD4B was upregulated by upstream mutations in NOTCH1 and NOTCH2. SAMD4B could affect the instability of APOA2 mRNA and inhibit APOA2 through RNA modification. Among many immune checkpoints, only PD-L1 expression was consistent with APOA2 expression, and they both had a direct interaction. The PPAR signalling pathway was significantly enriched in the high-SAMD4B-expression group, and CD29 showed a significant positive correlation with PDCD1, which suggested that CD29+CD8+ cells might participate in this immune regulation pathway. Fewer CD29+CD8+ T cells were found in high-SAMD4B-expression patients, who had a better prognosis, thus indicating that SAMD4B improved the immune microenvironment and weakened the immune escape of tumour cells from naive CD29+CD8+ T cells (Supplementary Fig. 13).

In conclusion, this study is the first to propose a TCC treatment strategy, provide evidence that the TCC therapy was associated with a better prognosis in the clinical treatment of patients with advanced HCC than other treatments, which exert an efficient anti-HCC effect by inducing the SAMD4B-APOA2 axis to inhibit tumour immune evasion.

## Materials and methods

### Sample collection

We retrospectively collected the clinical data of patients with unresectable HCC diagnosed at Zhongshan Hospital, Fudan University, from May 2017 to May 2020. Sixty-five patients were in the control cohort, and 27 patients were in the study cohort. All patients were initially treated with TACE, while patients in the study group were also administered a combination of oral medications daily, consisting of thalidomide (50 mg once every night), carmofur (100 mg three times a day) and cantharidin (compound Mylabris capsules 750 mg twice a day), named as TCC cocktail or thalidomide based cocktail (TBC) until tumour progression or the development of intolerable side effects.

Cohort 2, named the HCC retrospective cohort, consisted of 158 patients, and cancer and adjacent tissues were collected for immunohistochemical staining assays.

The ZS-SEQ-HCC cohort consisted of 30 patients with transcriptomics and whole-exome capture sequencing analysis data, which had been uploaded to the National Center for Biotechnology Information (NCBI) (https://www.ncbi.nlm.nih.gov/) with accession numbers GSE195952 and PRJNA802641.

Two public datasets were downloaded from NCBI: a breast cancer dataset (PRJNA734812) and a colon cancer dataset (GSE178341). The breast cancer dataset contained 26 primary pretreatment tumours, including 11 ER+, 5 HER2+ and 10 TNBC+ tumours; the colon cancer dataset contained 62 samples.

### Statistical analysis

Statistical analyses were performed using GraphPad Prism (version 8.0) for experimental data and R (v.4.1.0) for sequencing data. Comparisons between groups were conducted using Fisher’s exact test for categorical variables. Student’s *t* tests and Wilcoxon rank-sum tests were used for continuous variables. Comparisons among multiple groups were made by one-way ANOVA and Tukey’s post hoc test. Survival analysis was conducted using log-rank tests. *P* < 0.05 was considered to be statistically significant.

### Supplementary information

Supplementary Information

Supplementary Data 1

Supplementary Data 2

Supplementary Data 3

Supplementary Data 4

original data

## Data Availability

The original data and processed scRNA-seq data of 6 PDX samples and 4 HCC samples have been uploaded to the National Center for Biotechnology Information (NCBI) Gene Expression Omnibus (https://www.ncbi.nlm.nih.gov/geo/), with an accession number of GSE222791. The transcriptomics data and metabolomics data of the ZS-SEQ-HCC cohort have been uploaded to the NCBI Gene Expression Omnibus (https://www.ncbi.nlm.nih.gov/geo/), with accession number GSE195952. All data in the main text and supplementary materials are available upon request
