# Author Correction: Synergistic immunochemotherapy targeted SAMD4B-APOA2-PD-L1 axis potentiates antitumor immunity in hepatocellular carcinoma

**DOI:** 10.1038/s41419-024-06877-2

**Published:** 2024-07-17

**Authors:** Feng Qi, Jian Zhang, Jia Li, Donghe Li, Na Gao, Zhuoran Qi, Xiuyan Kong, Zhijie Yu, Yuan Fang, Wenguo Cui, Jinglin Xia

**Affiliations:** 1 National Medical Center & National Clinical Research Center for Interventional Medicine. Liver Cancer Institute, Zhongshan Hospital, Fudan University, 180 Fenglin Road, Shanghai, 200032 China; 2 Department of Oncology, Ruijin Hospital, Shanghai Jiao Tong University School of Medicine, Shanghai, 200025 China; 3 Department of Thoracic Surgery, Ruijin Hospital, Shanghai Jiao Tong University School of Medicine, Shanghai, 20025 China; 4 Shanghai Ji Ai Genetics and IVF Institute, Obstetrics and Gynecology Hospital, Fudan University, Shanghai, 200011 China; 5 Department of Laboratory Medicine, Zhongnan Hospital of Wuhan University, Wuhan University, Wuhan, 430071 China; 6 Zhejiang Key Laboratory of Intelligent Cancer Biomarker Discovery and Translation, First Affiliated Hospital of Wenzhou Medical University, Wenzhou, 325035 China; 7 Department of Liver Surgery, Key Laboratory of Carcinogenesis and Cancer Invasion (Ministry of Education), Liver Cancer Institute, Zhongshan Hospital, Fudan University, Shanghai, 200032 China; 8 Department of Orthopaedics, Shanghai Key Laboratory for Prevention and Treatment of Bone and Joint Diseases, Shanghai Institute of Traumatology and Orthopaedics, Ruijin Hospital, Shanghai Jiao Tong University School of Medicine, Shanghai, 200000 China

**Keywords:** Cancer microenvironment, Targeted therapies

Correction to: *Cell Death & Disease* 10.1038/s41419-024-06699-2, published online 17 June 2024

In this article

1. On Page 1 : Remove the email icon for author Feng Qi and update email addresses from “email:qf12486@rjh.com.cn; fangyuan1029@126.com;wgcui80@hotmail.com;xiajinglin@fudan.edu.cn” to “email:xiajinglin@fudan.edu.cn;wgcui80@hotmail.com;fangyuan1029@126.com”.
2. On Page 14, in the “Additional Information” section: Revise “Correspondence and requests for materials should be addressed to Feng Qi, Wenguo Cui or Jinglin Xia” to “Correspondence and requests for materials should be addressed to Jinglin Xia, Wenguo Cui or Yuan Fang”.
3. On Page 2, Results section, third paragraph: Correct “In colony formation assays, CAN decreased the viability of MHCC97-L, MHCC-LM3, and HuH-7 cells, which was expected because CAN is the precursor of 5-FU, which has been demonstrated to have antitumour effects [13]. However, the combination of THA and CAN had no further impact on cell viability in any of the six types of HCC cells (Fig. 1j, k). Furthermore, increasing the concentration of THA, CAN, or THA+CAN also had a limited effect on the cell survival rate (Supplementary Fig. 1a–f).” to “In colony formation assays, CAR decreased the viability of MHCC97-L, MHCC-LM3, and HuH-7 cells, which was expected because CAR is the precursor of 5-FU, which has been demonstrated to have antitumour effects [13].
  However, the combination of THA and CAR had no further impact on cell viability in any of the six types of HCC cells (Fig. 1j, k). Furthermore, increasing the concentration of THA, CAR, or THA+CAN also had a limited effect on the cell survival rate (Supplementary Fig. 1a–f).”
4. On Page 3, in the legend for Fig. 1c: Correct “AFP and PIVKA-II expression were significantlyd in the study cohort.“ to “AFP and PIVKA-II expression were significantly decreased in the study cohort.”.
5. On Page 3, in the legends for Fig. 1d: Correct “d After patient #1 underwent 5 courses of TCC therapy, a significant decrease in tumour number and remarkable tumour volume shrinkage were found by CT scan.” to “d Patient #1 underwent 5 courses of TCC therapy, a significant decrease in tumour number and remarkable tumour volume shrinkage were found by CT scan. “
6. On Page 3, in the legends for Fig. 1e : Correct “e After patient #2 underwent 3 courses of TCC therapy, remarkable tumour volume shrinkage was found by CT scan.” to “e Patient #2 underwent 3 courses of TCC therapy, remarkable tumour volume shrinkage was found by CT scan.”

The original article has been corrected.

