# Supplementary material for: Synergistic immunochemotherapy targeted SAMD4B-APOA2-PD-L1 axis potentiates antitumor immunity in hepatocellular carcinoma
Source: Cell Death Dis. 2024 Jun 17;15(6):421. doi: 10.1038/s41419-024-06699-2 (PMC11183041; doi:10.1038/s41419-024-06699-2)
Supplement: Supplementary file 1 — Supplementary Information [file 41419_2024_6699_MOESM1_ESM.docx]

**Supplementary information for the manuscript**

**Synergistic immunochemotherapy targeted SAMD4B-APOA2-PD-L1 axis potentiates antitumor immunity in hepatocellular carcinoma**

Feng Qi, Jian Zhang, Jia Li, Donghe Li, Na Gao, Zhuoran Qi, Xiuyan Kong, Zhijie Yu, Wenguo Cui and Jinglin Xia

Correspondence: Jinglin Xia, [xiajinglin@fudan.edu.cn](mailto:xiajinglin@fudan.edu.cn); Wenguo Cui, [wgcui80@hotmail.com](mailto:wgcui80@hotmail.com); Feng Qi, [qf12486@rjh.com.cn](mailto:qf12486@rjh.com.cn).

**Supplementary Materials and Methods**

**PDX mouse models and drug treatment**

We isolated fresh tumour tissues from HCC patients. We anaesthetized NOG mice and subcutaneously implanted HCC tissues into the right superior flank of the mice. After two months, when the diameter of the tumours reached 1 cm, the subcutaneous PDX tumours were removed, dissected into 3 pieces approximately 2 × 2 × 2 mm in size, and retransplanted into the flanks of nude mice for approximately 30 days for growth. The mice were euthanized at no more than 5 weeks or at the time when tumours reached 10 mm in diameter. For the drug treatment groups, the mice received 18 mg/kg of thalidomide, 225 mg/kg of carmofur, 45 mg/kg of cantharidin, TC (thalidomide + carmofur) or TCC (thalidomide + carmofur + cantharidin) per mouse per kilogram every two days via tail intravenous injection on the eighth day after transplantation. For the control group, the mice received isovolumetric DMSO per mouse per kilogram every two days via tail intravenous injection on the seventh day after transplantation.

**Colony formation assays**

Colony formation assays were used to evaluate cell proliferation. A total of 2000 cells/well were plated in a 6-well plate. After 2 weeks of culture, the cells were fixed with parachrome and stained with crystal violet. Photographs were taken, and data were collected.

**10x Single-cell isolation**

Fresh tumour and adjacent normal tissues were surgically removed from patients, immersed in complete medium containing 90% Dulbecco’s modified Eagle medium (DMEM; Cat# 11054001, Gibco) and 10% foetal bovine serum (FBS; Cat# 16140071, Gibco), and transported to the laboratory in a refrigerated container. Suitable small tissue blocks (no necrotic foci/no haemorrhagic foci/less fibrous connective tissue) were cut into pieces (diameter 1-3 mm). The pieces were transferred to gentleMACS C Tubes (Cat# 130-096-334, Miltenyi Biotec), with 5 mL of digestive enzyme included in the Tumour Dissociation Kit (Cat# 130-095-929, Miltenyi Biotec). Then, the tissues were made into a single-cell suspension using the gentleMACS Dissociator (Cat# 130-093-235, MiltenyiBiotec) following these steps: milling; incubation at 37°C for 30 min on a shaker; milling; incubation at 37°C for 30 min; milling; and filtering through a 70 mm filter, plus 2% FBS. Finally, the single-cell suspension was centrifuged at 400 × g for 7 mins, resuspended in CELLSAVING (Cat# C40050, NCM Biotech), cryopreserved in cryotubes (Cat# 430488, Corning) and then stored at -80℃.

For paired validation cohort 1, single cells or single nuclei (sn) were used for scRNA-seq or snRNA-seq. The preparation of a single-cell suspension was the same as described in the last paragraph. Single nuclei were prepared as follows: frozen tumour tissue pieces were placed in 1 mL homogenization buffer (prechilled) containing 10 mM Tris pH 8.0 (Cat# AM9856, Ambion), 250 mM sucrose (Cat# V900116-500G, Sigma‒Aldrich), 25 mM KCl (Cat# AM9640G, Ambion), 5 mM MgCl2 (Cat# AM9530G, Ambion), 1% bovine serum albumin (BSA; Cat# C102301, Sangon Biotech), 0.1% NP-40 (Cat# 11754599001, Roche), 0.2 U/µl RNasin Plus (Cat# N2611, Promega), 13 protease inhibitor (Cat# G6521, Promega), and 0.1 mM DTT (Cat# 707265ML, Thermo Fisher Scientific) in a 2 mL Dounce homogenizer (Cat# D8938, Sigma-Aldrich) for 5 minutes. The tissue was then homogenized by 10 strokes of the loose pestle and 10 strokes of the tight pestle. The Dounce homogenizer was submerged in ice during the grinding step. One millilitre of homogenization buffer was added to the Dounce homogenizer, and then the homogenate was passed through a 40 mm cell strainer (Cat# 43-10040-70, Miltenyi Biotec) into a 15 mL conical tube and centrifuged at 300 × g for 5 mins. Sediment was resuspended in 1.5 mL Blocking Buffer containing 1/3x phosphate buffered saline (PBS; Cat# 10010049, Thermo Fisher Scientific), 1% filtered sterilized BSA, and 0.2 U/ml Rnasin Plus by pipetting up and down gently on ice and centrifuged at 300 × g for 5 mins. The previous step was repeated once. Nuclei were resuspended in 0.04% BSA in PBS, stained with 40-60-diamidino-2-phenylindole (DAPI; Cat# D9542, Sigma‒Aldrich) for counting and diluted to an appropriate concentration.

This process was based on the microfluidic platform Chromium ™ Single cell 3'Solution. The primers were composed of full-length Illumina TruSeq Read 1 sequencing primer, 16 bp 10X barcode sequence (used to distinguish cells), 12 bp unique molecular identifier (UMI) (to distinguish different transcripts of unified cells and remove PCR duplications), and 30 bp poly dT reverse transcription primer. The prepared cell suspension, 10X barcode gel magnetic beads and oil drops were added to different channels (8 channels in total) of Chromium Chip B, and GEM was formed through the microfluidic "double cross" system. To obtain a single-cell reaction system, the concentration of the cell suspension was controlled at 700-1200 cells/µl, so 90-99% of GEM produced did not contain cells, and most of the remaining GEM contained one cell.

**Single-cell sorting, reverse transcription, amplification and sequencing**

Briefly, the following steps were used to prepare cDNA products: dispensing lysis buffer and single cells into a 5,184 microwell chip, cell lysis, reverse transcription, cDNA amplification and identification of single-cell wells based on qPCR results, product extraction, fragmentation and PCR, and then cyclization into ssDNA libraries. Each single-cell library was labelled with a barcode in the PCR step and then sequenced on a BGISEQ500 sequencer with 100-bp single-end reads. For validation cohort 1, single-cell or single-nucleus transcriptome amplifications, cDNA libraries and TCR VDJ libraries were prepared using the 10x Genomics Chromium Single Cell 50 Library Construction Kit (Cat# 1000020, 10x Genomics) and Chromium Single Cell V(D)J Enrichment Kit, Human T-Cell (Cat# 1000005, 10x Genomics) following the manufacturer’s instructions. Briefly, these steps included cDNA amplification, enrichment of the TCR VDJ fragment twice from the cDNA product and library construction of cDNA or TCR VDJ. Finally, the libraries were cyclized into ssDNA libraries with the MGIEasy Circularization Kit (Item No. 1000005259, MGI). The cDNA libraries were sequenced on the BGISEQ500 sequencer with 8+26+100-bp pair-end reads, and the TCR VDJ libraries were sequenced on the BGISEQ500 sequencer with 8+150+150-bp paired-end reads.

**Bulk DNA and RNA extraction and sequencing**

Genomic DNA and RNA of tissue samples were extracted using the QIAamp AllPrep DNA/RNA Mini Kit (80204, QIAGEN) according to the manufacturer’s specifications, including the steps of DNA/RNA adsorption, purification, and collection in columns.

The concentrations of DNA were quantified using a Qubit dsDNA BR Assay Kit (Thermo Fisher Scientific), and the quality of the DNA was evaluated by agarose gel electrophoresis. The exome libraries were constructed using the MGIEasy Exome Universal Library Prep Set (MGI) according to the instructions. In brief, DNA was fragmented, followed by adaptor ligation at both ends and PCR amplification. Samples were sequenced on a BGISEQ500 sequencer with 100-bp paired-end reads. In addition, multiplex PCR was designed to amplify the CDR3 regions of the rearranged TCR b chain (TRB) from genomic DNA. The enriched TRB products were cyclized into ssDNA libraries by the MGIeasy Circularization Kit and then sequenced on the BGISEQ500 sequencer with 100-bp single-end reads.

**10X Single-cell analysis**

We used the Seurat R package (v4.1.3) to perform unsupervised clustering of the single cells using the read count matrix as input. First, the read counts for each cell were divided by the total counts for that cell, multiplied by the scale factor (10,000), and then natural-log transformed. We performed principal component analysis (PCA) on the normalized expression matrix using highly variable genes identified by the ‘‘SCTransform’’ function.

The Harmony R package “RunHarmony” was used to remove the batch effect between samples. Following the results of Harmony, the appropriate principal components (Harmonys) were selected for clustering with the specific resolution parameters. Finally, to detect cluster-specific expressed genes, the clusters were compared pairwise using the Seurat ‘‘FindAllMarkers’’ function to test genes with > 0.25-fold difference (log-scale) on average between the two groups of cells.

For the clustering of all cells, the top 25 Harmonys were selected with a resolution parameter equal to 0.6. For the clustering of T lymphocytes, the top 20 PCs were selected with a resolution parameter equal to 1.

Marker genes for each cluster were identified with the Wilcoxon rank-sum test with default parameters via the FindAllMarkers function in Seurat. Differential expression analysis was carried out using the ‘‘FindMarkers’’ function, implemented in the Seurat package, with log-scaled fold change >=0.25 and P value <= 0.05 (Wilcoxon rank sum test) CD8 cell developmental trajectory.

**Immunohistochemistry**

Human and mouse cancer tissues were first embedded in paraffin and then sectioned into 4 μm slices. The sections were deparaffinized and rehydrated in a descending ethanol series. Following antigen retrieval, the sections were incubated with 3% hydrogen peroxide for 20 min. Tissue slides were then incubated overnight at 4°C with the following primary antibodies: anti-SAMD4B antibody (1:100; Proteintech; Cat No. 17723-1-AP), anti-APOA2 antibody (1:400; Abcam; Cat No. ab92478), anti-PD-L1 antibody (Cell Signaling Technology, Cat No. 41726), anti-Notch1 antibody (Abcam; Cat No. ab52627) and anti-Notch2 antibody (Abcam; Cat No. ab118824). A two-step immunohistochemical staining kit (zsbio; Cat No. PV-9000) was used for protein expression analysis according to the manufacturer’s instructions. Finally, the slides were visualized with 3,3′-diaminobenzidine solution (DAB) and counterstained with haematoxylin. At least five fields were reviewed for each slide at 400× magnification by two independent investigators in a randomized, double-blind manner. Immunoreactivity was semiquantitatively scored according to the following scale: 0, < 5% immunoreactive cells; 1, 5-25% immunoreactive cells; 2, 25-50% immunoreactive cells; 3, 50-75% immunoreactive cells; and 4, > 75% immunoreactive cells. Staining intensity was also semiquantitatively scored as 0 (negative), 1 (weak), 2 (intermediate), or 3 (strong). The final score for each patient was expressed as the product of the proportion and intensity scores. The cut-off scores for high and low expression were ≥ the median or < the median, while those for positive and negative expression were ≥ 4 or < 4.

**Transfection of plasmid or small interfering RNA (siRNA)**

Experimental plasmids or small interfering RNAs (siRNAs) were purchased from GeneChem: SAMD4B plasmid, APOA2 plasmid, PD-L1 plasmid, PD-L2 plasmid, FGL1 plasmid, HMGB1 plasmid and siSAMD4B-1, siSAMD4B-2. The plasmids or siRNAs were transfected into HEK293T cells. Transfection efficiency was validated using qRT‒PCR.

**RNA isolation, reverse transcription, and quantitative real-time polymerase chain reaction (qRT‒PCR)**

RNA was extracted from HCC cell lines and frozen tumour samples. Complementary DNAs were synthesized using the PrimeScript Reverse Transcriptase Kit (Takara, Osaka, Japan). The ABI PRISM 7900 Sequence Detection System (Applied Biosystems) was used for amplification and detection. GAPDH was the endogenous control. Gene levels were normalized to GAPDH to generate a 2-ΔΔCt value for the relative expression of each transcript.

**Coimmunoprecipitation (co-IP) and Western blot analysis**

To immunoprecipitate ectopically expressed Flag, Myc or HA-tagged proteins, transfected cells were lysed 24 h posttransfection in BC100 buffer. Whole-cell lysates were immunoprecipitated with monoclonal anti-Flag, anti-Myc or anti-HA antibody-conjugated agarose beads (Sigma‒Aldrich) at 4°C overnight. After three washes with lysis buffer, followed by two washes with BC100 buffer, the bound proteins were eluted using Myc, Flag or HA-Peptide (Sigma‒Aldrich) prepared in BC100 for 3 h at 4°C. The eluted protein sample was resolved by SDS–PAGE. Cells cultured in androgen-depleted medium were lysed in modified binding buffer (50 mM Tris-HCl pH 7.5, 150 mM NaCl, 1% NP-40, 0.1% SDS and 1% protease inhibitor cocktails). Cell lysates were incubated with biotin-labelled and streptavidin beads at 4°C for 12 h. The beads were washed in wash buffer (50 mM Tris, pH 7.4; 150 mM NaCl; 0.05% Nonidet P-40 (NP-40); 1 mM MgCl2) at 4°C six times. Briefly, protein samples were denatured, subjected to SDS-polyacrylamide gel electrophoresis (SDS/PAGE) and transferred to nitrocellulose membranes (Bio-Rad). The membranes were immunoblotted with specific primary antibodies and horseradish peroxidase-conjugated secondary antibodies and visualized by SuperSignal West Pico Stable Peroxide Solution (Fisher).

**Multiplex immunofluorescence staining**

We used antibodies specific for SAMD4B (Proteintech, Cat No. 17723-1-AP), CD3 (Abcam, Cat No. ab16669), CD4 (Abcam, Cat No. ab183685), CD8 (Abcam, Cat No. ab217344), CK18 (Abcam, Cat No. ab133263), CD29 (Proteintech, Cat No. 26918-1-AP), PD-L1 (Cell Signaling Technology, Cat No. 41726), and PD1 (Cell Signaling Technology, Cat No. 13684) for combined immunofluorescence (mIF) analysis. The sections were deparaffinated with xylene and then rehydrated with ethanol. We used Tris-EDTA buffer (pH 9.0) and incubated at the boiling point for 15 min for antigen repair and 3% hydrogen peroxide at room temperature for 15 min to block the activity of endogenous peroxidase. We used goat serum solution to block nonspecific antigens for 30 min. These sections were incubated overnight at 4°C with primary antibodies, and then the horseradish peroxide (HRP)-conjugated secondary antibody was added at room temperature for 30 min. Next, we incubated sections with Opal tyramide signal amplification (TSA) Fluorochromes (Opal 7-Colour Manual IHC Kit, Perkin Elmer, NEL811001KT) at 37°C for 20 min. Between each run, the Ab-TSA complex in sections was removed by microwaving, and sections were blocked with the goat serum solution. On the last run, 4′,6-diamidino-2-phenylindole, dihydrochloride (DAPI) was added for nuclei visualization and was mounted with glycerine.

**Orthotopic tumour model and drug treatment**

Hep1-6 cells were injected into mice [5×10^7^ cells suspended in 100 μL of serum-free Dulbecco’s modified Eagle’s medium (DMEM) and Matrigel (1:1; BD Biosciences, San Jose, CA), subcutaneous injection, right flank region]. Tumours were collected when they were 1 cm in diameter and cut into small pieces (2×2×2 mm3). The pieces were then transplanted into the livers of live mice (n=6 per group). The animals were euthanized 35 days after transplantation. For the drug treatment groups, the mice received 18 mg/kg of thalidomide, 225 mg/kg of carmofur, 45 mg/kg of cantharidin, TC (thalidomide + carmofur) or TCC (thalidomide + carmofur + cantharidin) per mouse per kilogram every two days via tail intravenous injection on the eighth day after transplantation. For the control group, the mice received isovolumetric DMSO per mouse per kilogram every two days via tail intravenous injection on the seventh day after transplantation.

**Flow cytometry analysis**

The markers of mouse T cells were determined by flow cytometry after staining with specific antibodies conjugated with different fluorescence. The following human or mouse antibodies were used: APC/CY7-anti-CD45, BV605-anti-CD3, FITC-anti-CD8, AF647-anti-CD4, and BV421-anti-CD29. All antibodies were purchased from Biolegend. The stained cells were analysed on a FACSCalibur flow cytometer (BD Bioscience), and data were analysed using FlowJo10 software (Tree Star, Inc.; Ashland; OR).

**RNA modification assay by LC‒MS/MS**

RNA modification assays by LC‒MS/MS were performed by CloudSeq Inc. First, 2 μg of RNA was digested into nucleosides with 2 U of nuclease P1 (Sigma, N8630) and 1.5 U of alkaline phosphatase (Sigma, P4252) at 37°C for 3 h. Then, 5 μL of the mixture solution was injected into an LC‒MS/MS system. The nucleosides were detected by an AB Sciex QTRAP 6500 LC‒MS/MS platform in positive ion mode.

**Nm-Seq (2′-O-Methylation) sequencing**

The Nm-Seq sequencing service was provided by Shanghai Yunxu Biotechnology Company, and its main process has been described previously (1). Briefly, the RNA samples were reacted with an RNA fragmentation reagent (GenSeq Inc.) at 95°C for 5 min. The fragmentation of RNA in thermal phosphatase (New England BioLabs) was end repaired at 37°C for half an hour. After repair, the RNAs were oxidized for half an hour in 10 mM sodium periodate solution at 37°C and then reduced using ethylene glycol. Then, shrimp alkaline phosphatase (New England BioLabs) was used to remove the end of the RNA phosphorus acid groups at 37°C for half an hour. The above oxidation‒reduction dephosphorization reaction was repeated 8 times. In the final round of the reaction, oxidation‒reduction only was performed, not the dephosphorization reaction. Then, mixed purification of RNA samples with the missing three 'phosphatase activity of T4 polynucleotide kinase (New England BioLabs) was performed at 37°C for 1 hour. Finally, the GenSeq ® Small RNA Library Prep Kit (GenSeq Inc.) agent box was used to build the sequencing library, the Qubit (Thermo Fisher) fluorescence method was used to quantify the library, and the Illumina HiSeq sequencing machine was used to perform sequencing.

**Supplementary Tables**

Supplementary Data 1. Clinicopathologic correlation and survival analysis of HCC patients from control cohort and study cohort.

Supplementary Data 2. Differential expression genes between among DMSO group, TC group, and TCC group in C16+C18.

Supplementary Data 3. Differential expression genes between DMSO group and THA group, between DMSO group and CAN group, and between DMSO group and CAR group in C16 + C18.

Supplementary Data 4. Clinicopathologic correlation and survival analysis of HCC patients with low and high expression of SAMD4B and APOA2 from tissue microarray (TMA) cohort.

**Supplementary Figures**


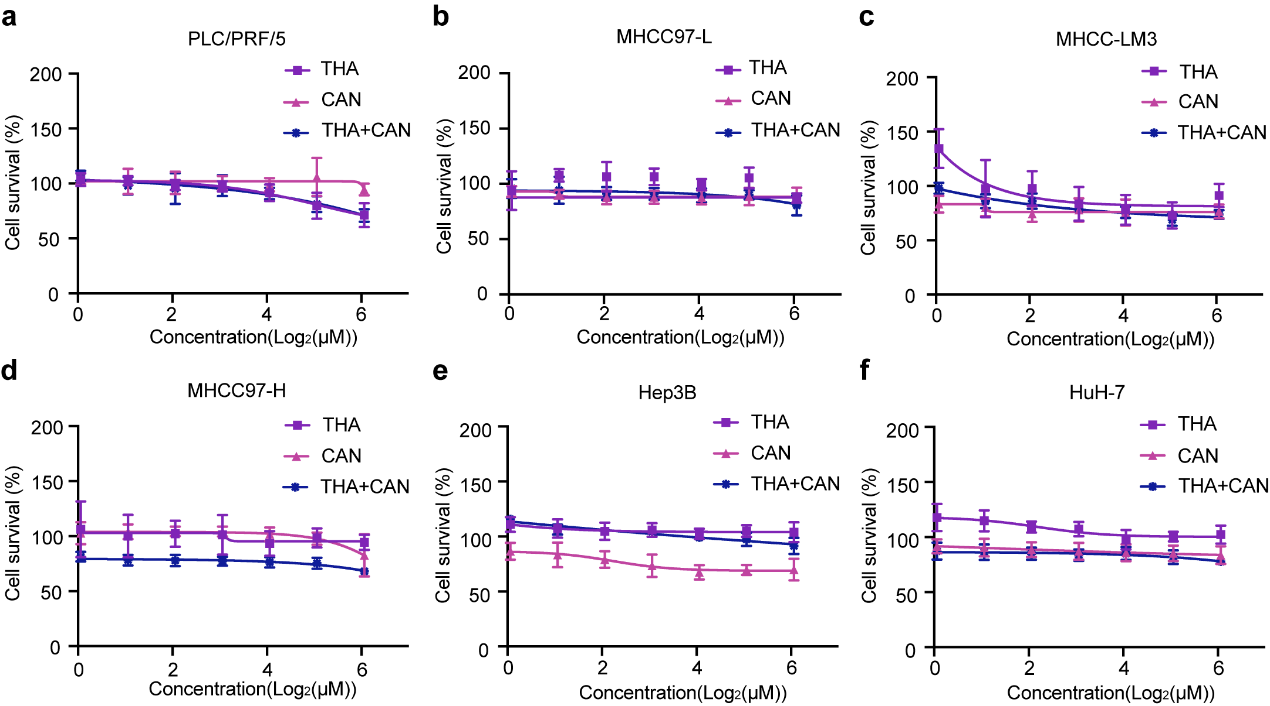


Figure S1. Increasing the concentration of THA, CAN, or THA+CAN had a limited effect on cell survival rate of 6 HCC cell lines.


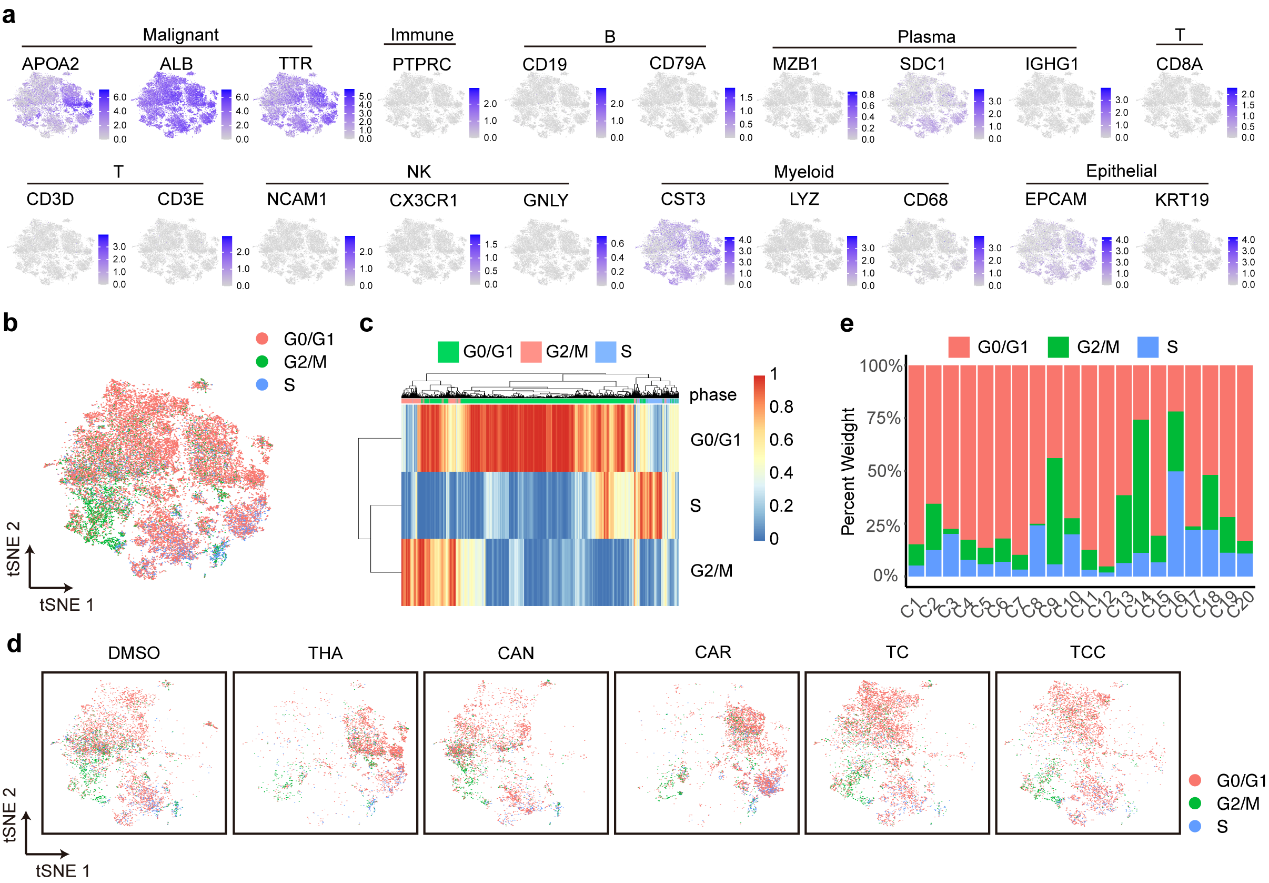


Figure S2. The immune cells identification and their cell cycle. (a) The immune cells were identified, including immune cells (B, plasma, T, NK and myeloid cells), and epithelial cells. The tSNE (b) and heatmap plots of the cell cycle of immune cells. (d) The tSNE plot of DMSO, THA, CAN, CAR, TC and TCC. (e) The cell cycle patterns of C16 and C18 were different from those of other clusters, with a higher proportion of G2/M and S phase cells.


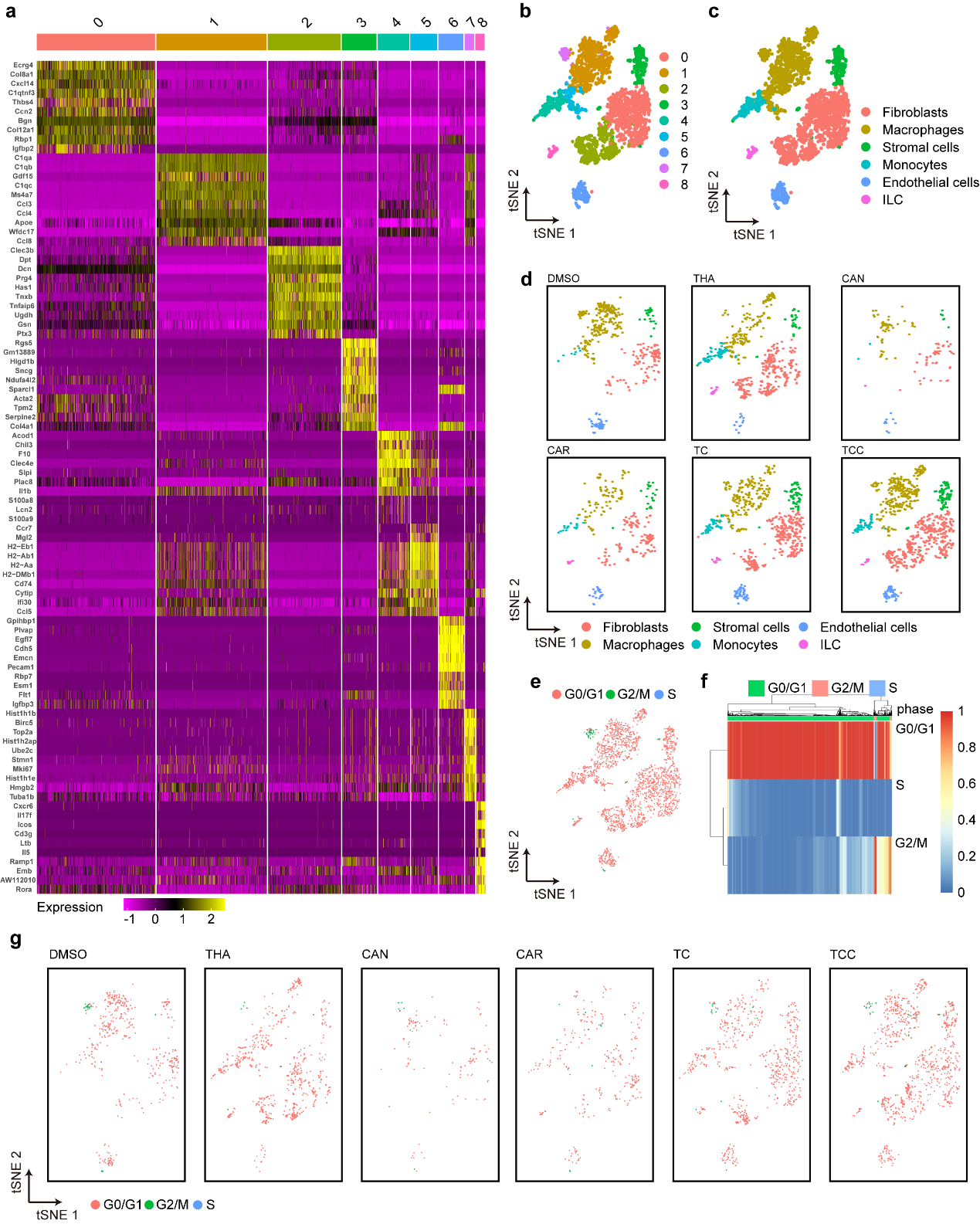


Figure S3. The cell clusters of murine-derived cells. The heatmap (a) and tSNE (b) plots of the murine-derived cells, which were represented by nine cell clusters. (c) and (d) These identified cell clusters could be assigned to known cell lineages through 6 marker genes. (e) The tSNE plot of cell cycle of murine-derived cells. (f) The heatmap plot of cell cycle of murine-derived cells. (g) The tSNE plot of cell cycle of murine-derived cells in single–agent and combined therapy.


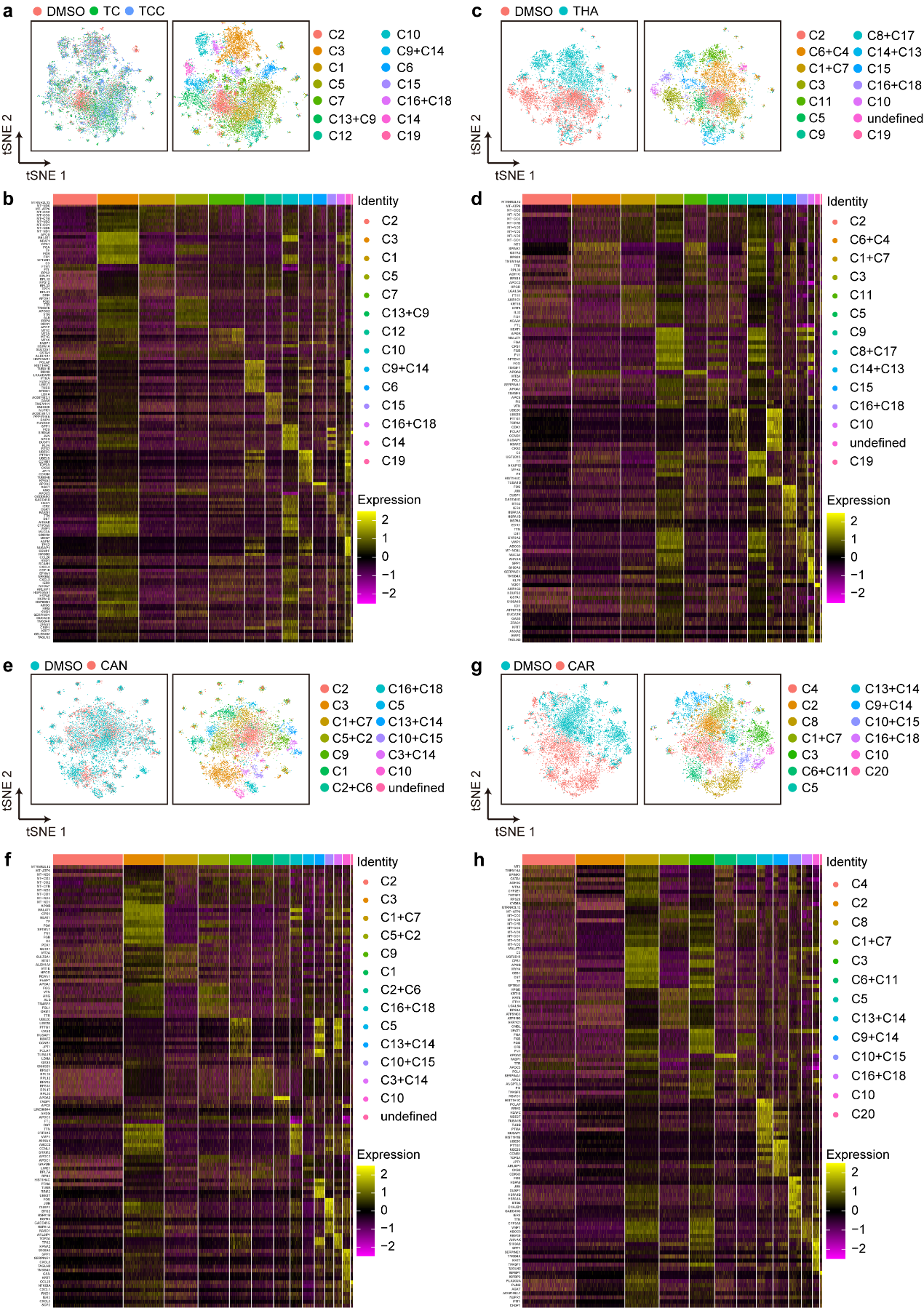


Figure S4. The results of DMSO group compared with single-agent and combined-agent groups separately. (a) and (b) The tSNE and heatmap of DMSO compared with TC and TCC. (c) and (d) The tSNE and heatmap of DMSO vs THA. (e) and (f) The tSNE and heatmap of DMSO vs CAN. (g) and (h) The tSNE and heatmap of DMSO vs CAR.


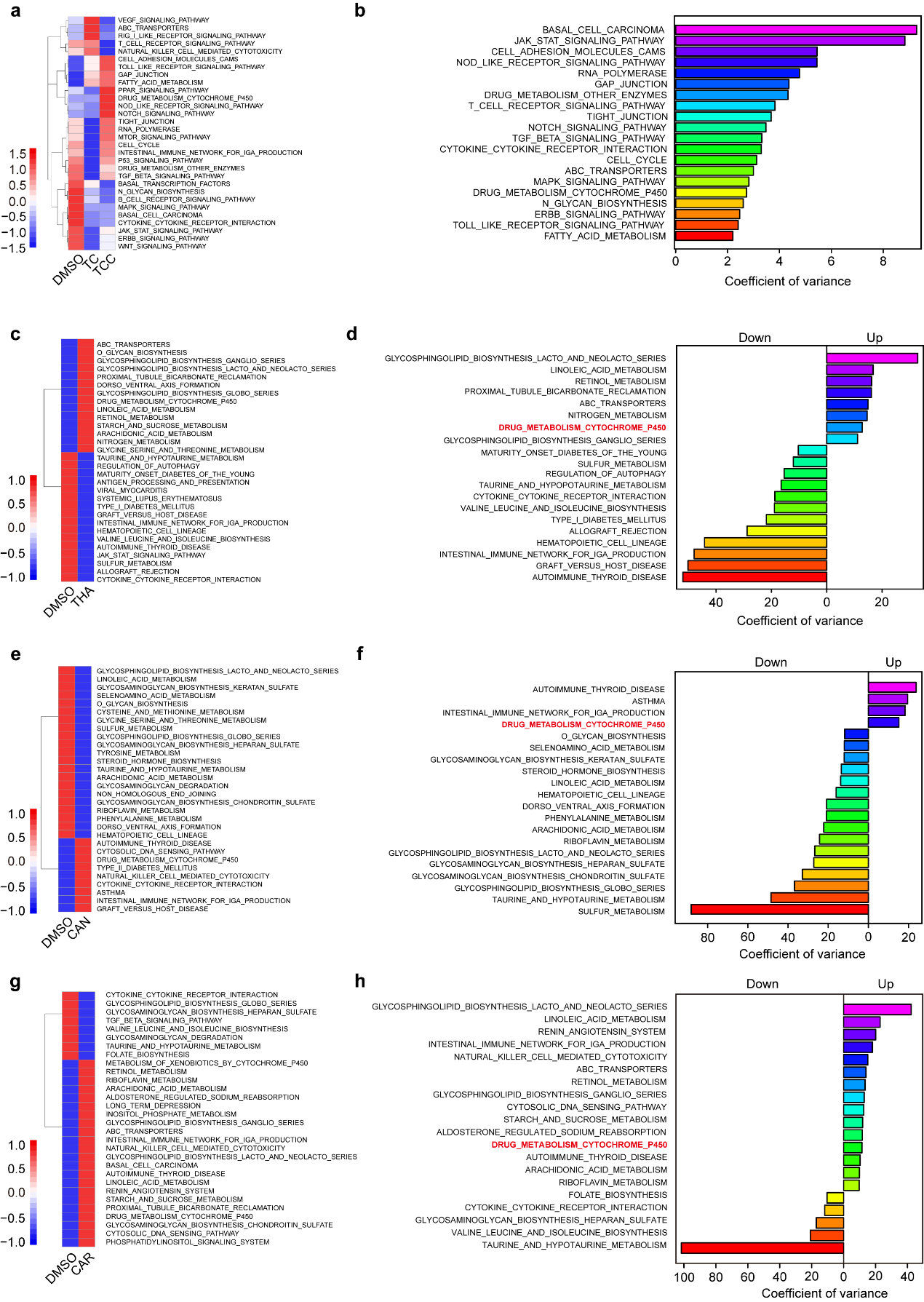


Figure S5. The functional analysis of DMSO group compared with single-agent and combined-agent groups separately. (a) and (b) The significantly different genes and pathways in DMSO compared with TC and TCC. (c) and (d) The significantly different genes and pathways in DMSO vs THA. (e) and (f) The significantly different genes and pathways in DMSO vs CAN. (g) and (h) The significantly different genes and pathways in DMSO vs CAR.


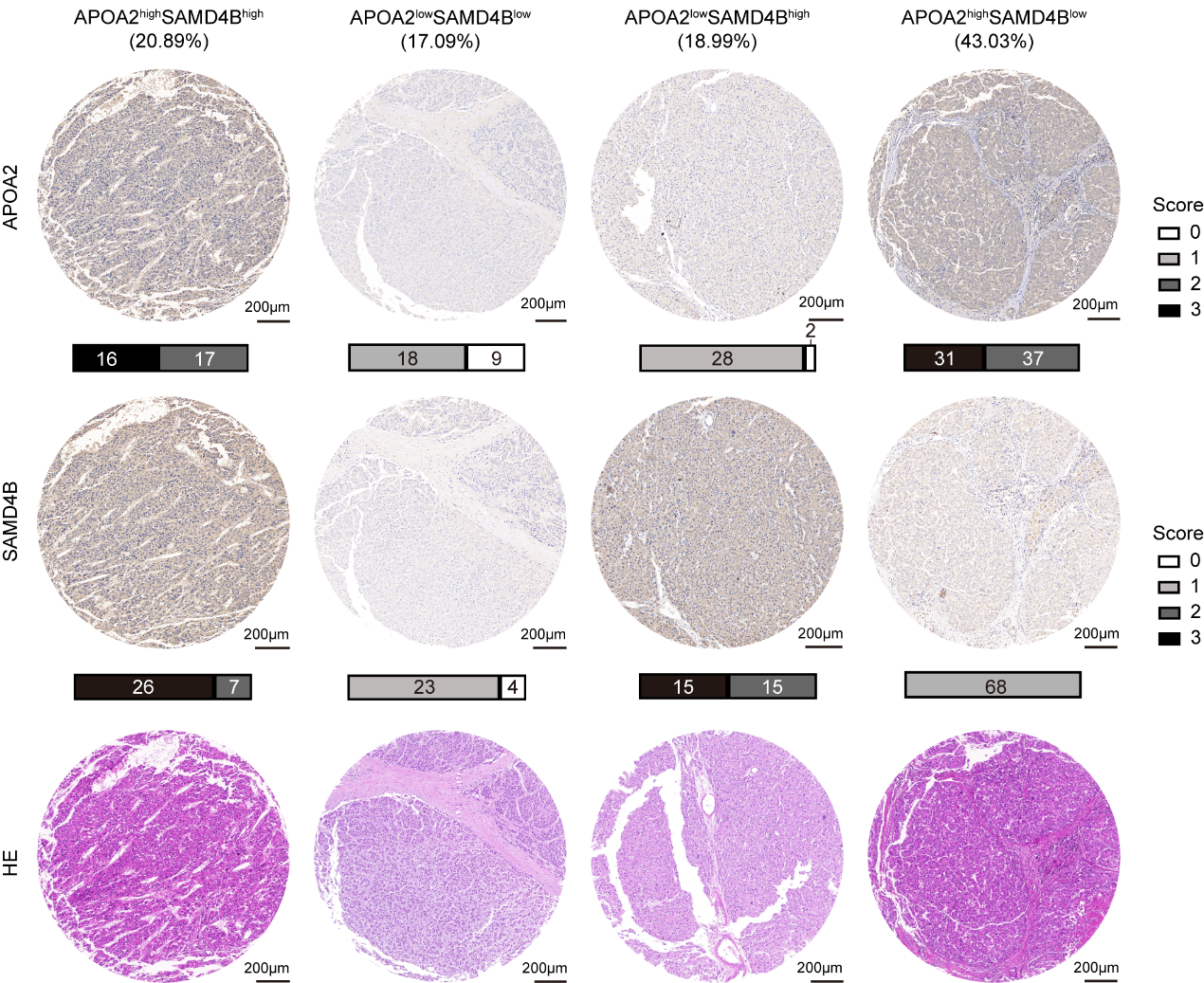


Figure S6. The immunohistochemical straining assays of the HCC retrospective cohort.


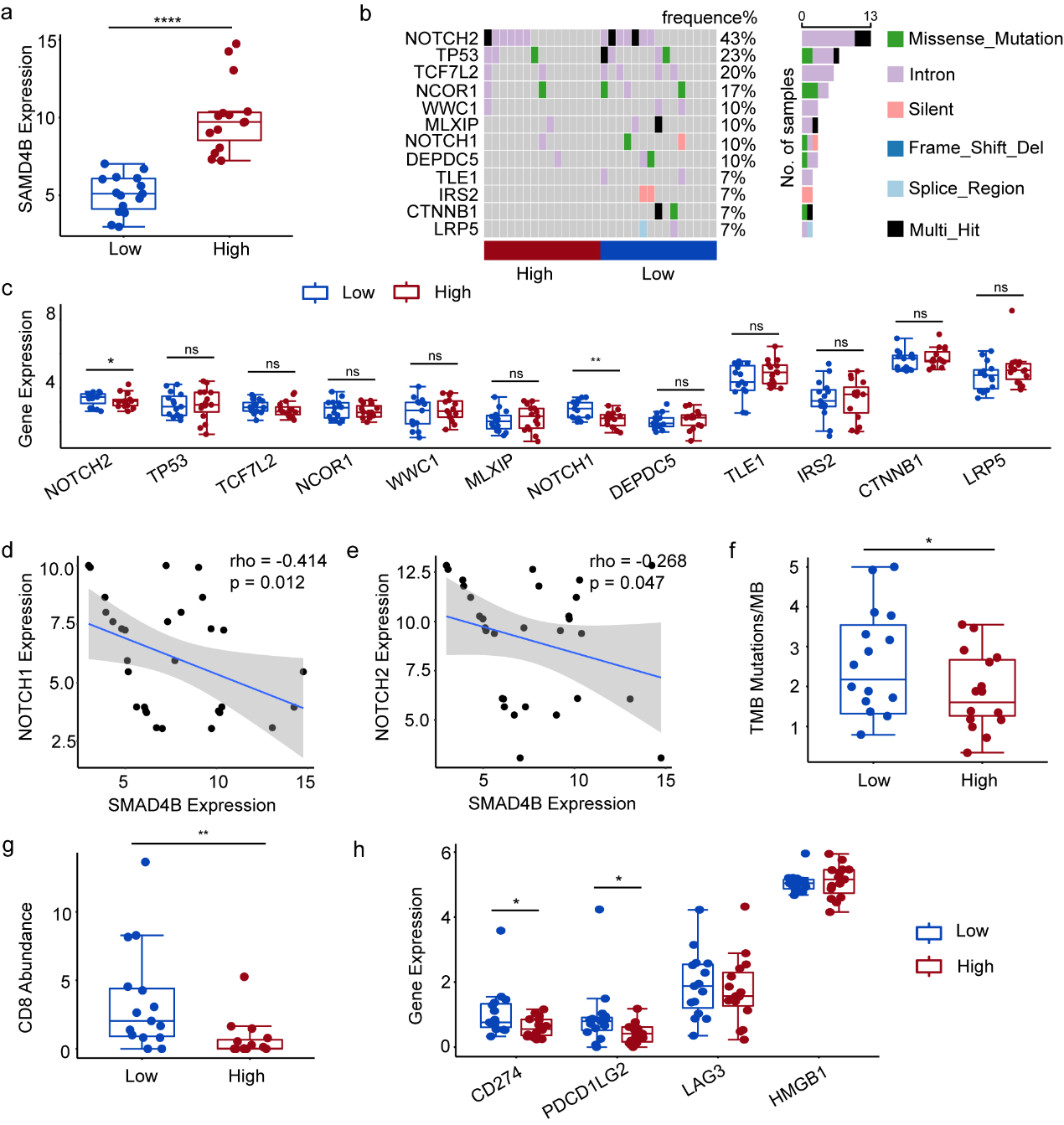


Figure S7. The potential upstream regulatory mechanism of SAMD4B by analyzing ZS-SEQ-HCC cohort. (a) The cohort was divided into high- and low-SAMD4B groups according to the expression of SAMD4B. (b) The top 12 genes with the highest mutation frequency. (c) NOTCH2 and NOTCH1 significantly expressed by comparing the low- and high-SAMD4B groups. (d) and (e) Both NOTCH2 and NOTCH1 were negatively correlated with SAMD4B. (f) Low-SAMD4B group exhibited a rather high TMB. (g) CD8 was significantly enriched in Low-SAMD4B group. (h) The level of SAMD4B expression could affect the immune checkpoints, such as CD274 and PDCD1LG2. Student’s test. (Level of significance: **, *P* < 0.01; *, *P* < 0.05; ns, *P* > 0.05).


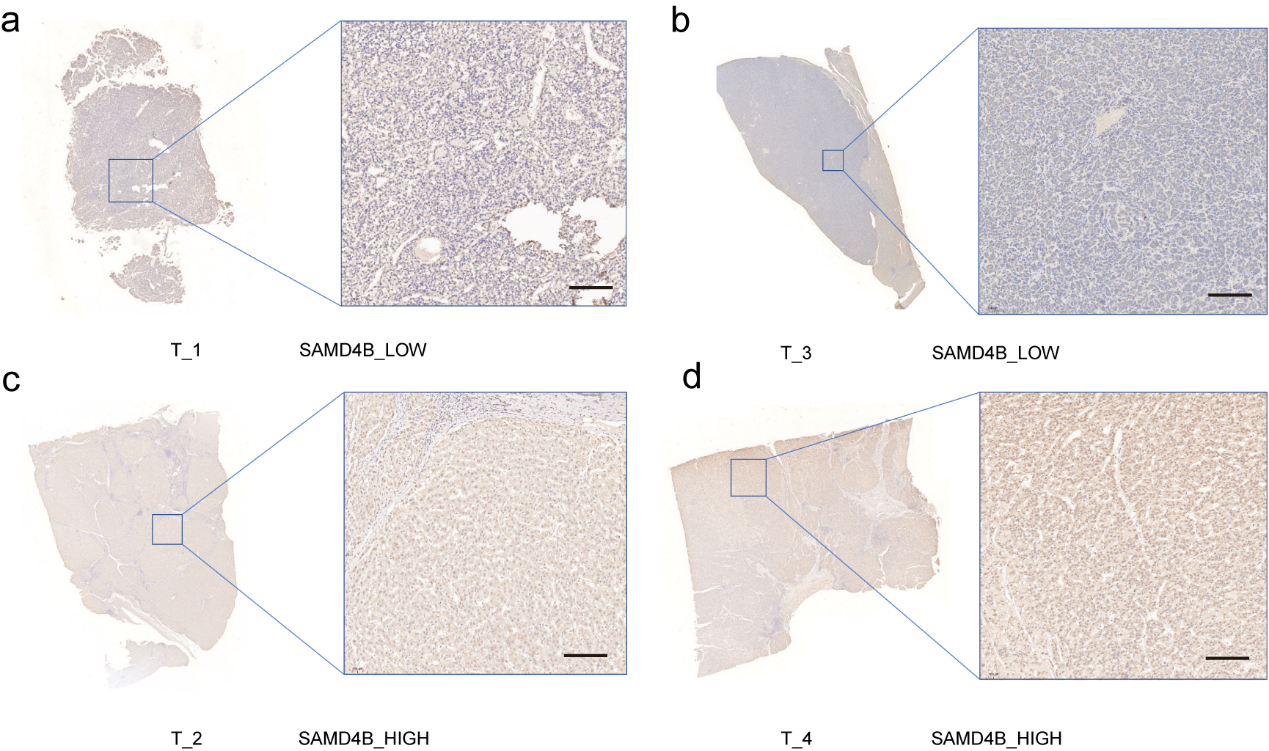


Figure S8. Immunohistochemical assays of four patients selected from low- (a, b) and high-SAMD4B groups (c, d).


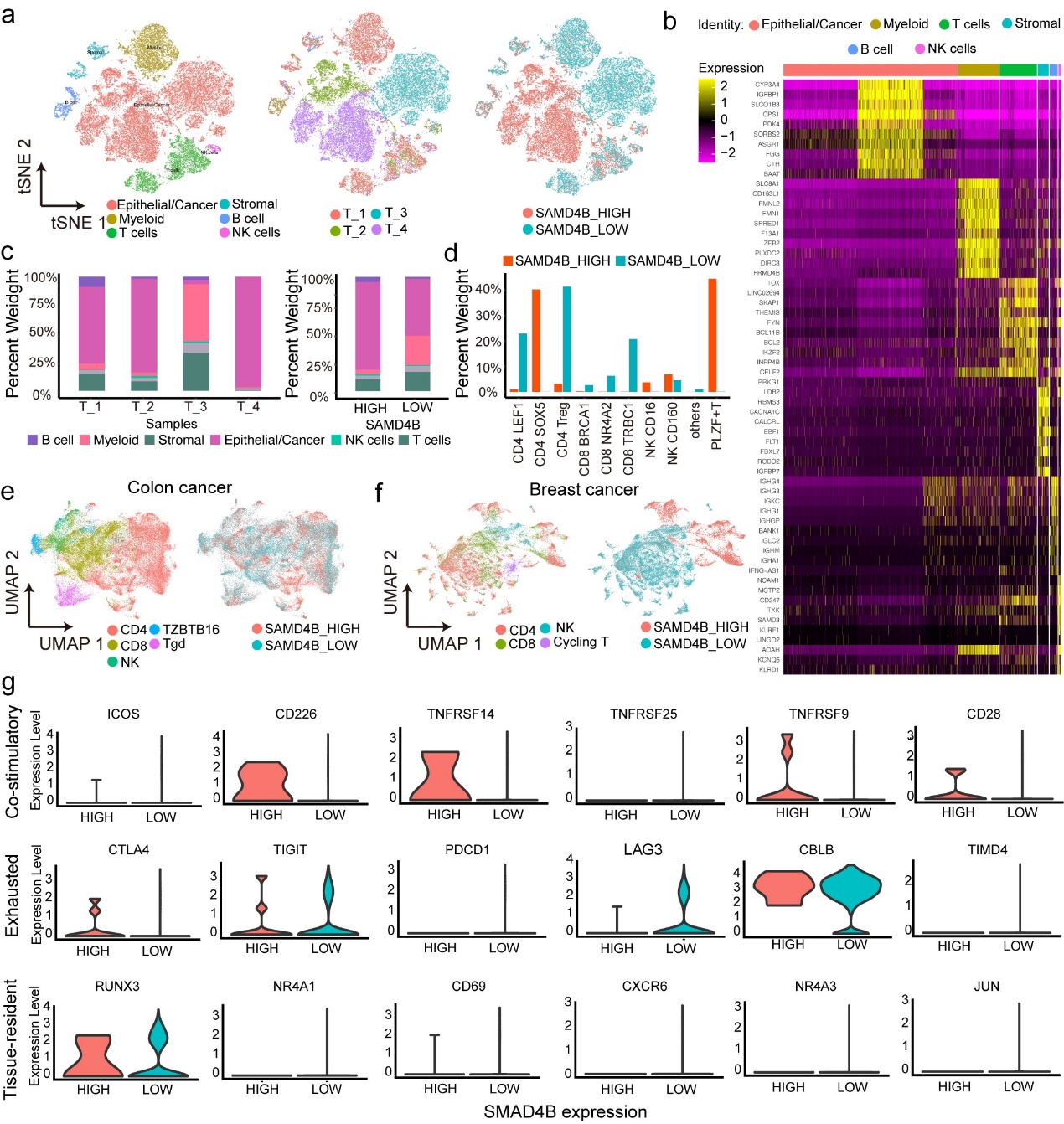


Figure S9. The heterogeneity of immune cell compositions among HCC tumors. (a) The tSNE plots of identified immune cells. (b) The heatmap plot of cell subtypes. (c) The proportions of these cell subtypes. (d) The proportions of unsupervised clustering of T cells and NK cells. The tSNE plots of identified cells in (d) breast cancer and (e) colon cancer. (g) The expression scores of co-stimulatory, exhausted and tissue-resident T cell phenotypes.


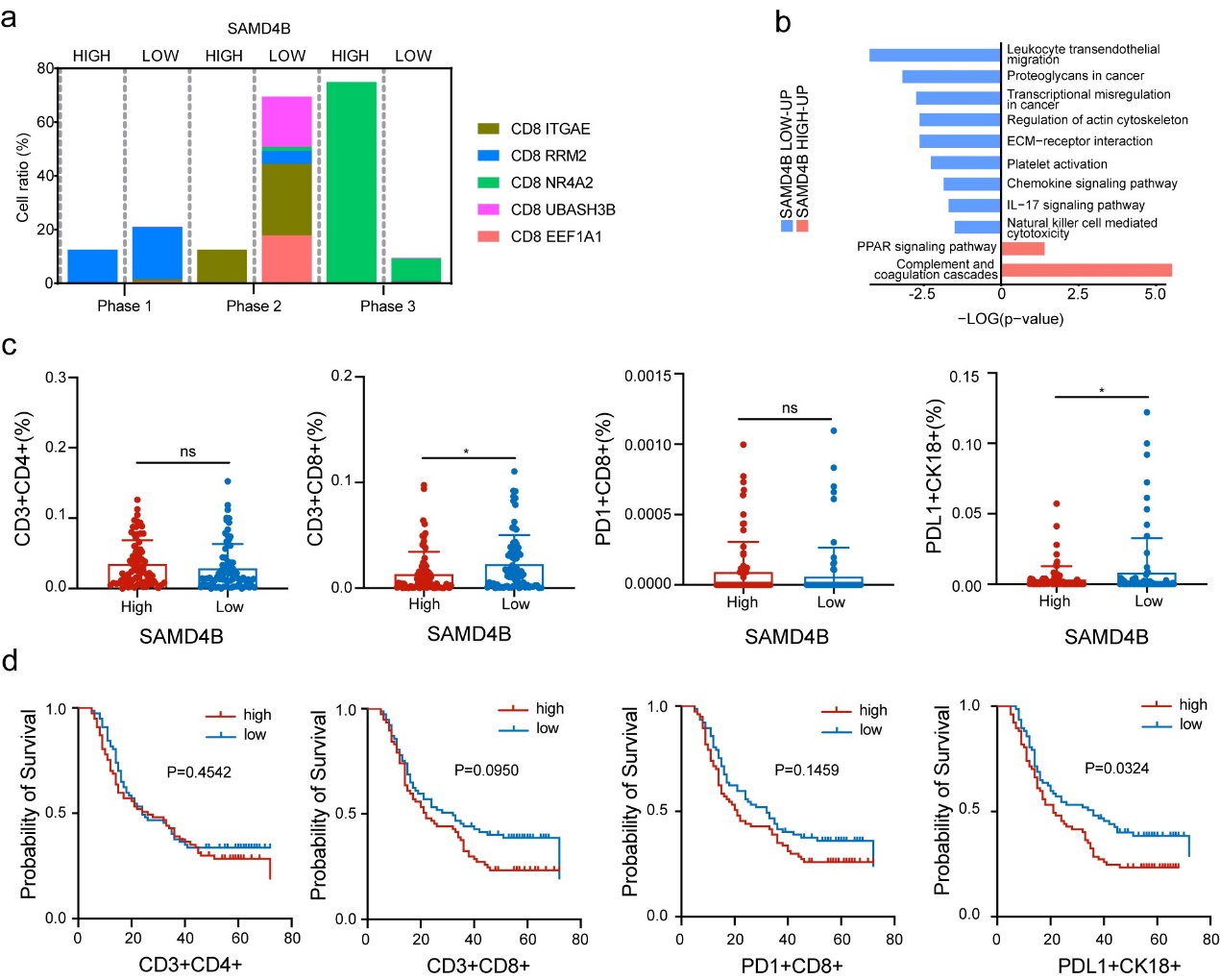


Figure S10. The unique transcriptional of CD8+ T cells in low-/high- SAMD4B groups. (a) CD8+ T cells in high-SAMD4B group were predominantly characterized as phases 3, representing the transition process from cytotoxic to exhausted states. (b) the PPAR signaling pathway significantly reduced in low-SAMD4B group. (c) The low-SAMD4B samples had more CD3+CD8+ T cells and higher expressed PD-L1+CK18+. (d) The prognosis of the expression level of CD3+CD4+, CD3+CD8+, PD1+CD8+ and PD-L1+CK18+ T cells. Student’s test. (Level of significance: *, *P* < 0.05; ns, *P* > 0.05).


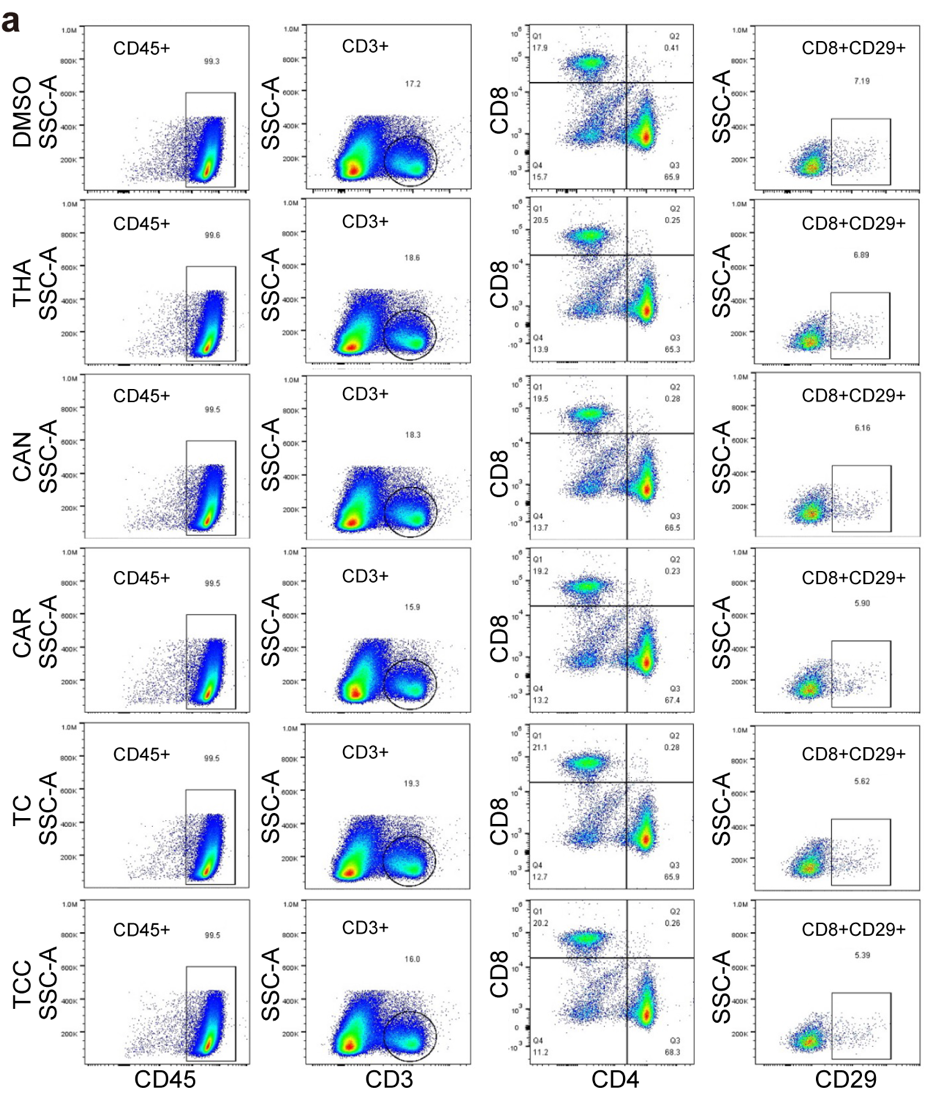


Figure S11. The utilized flow cytometry plot of the immunocompetent C57BL6/J mice, which were constructed orthotopic tumor and treated by TCC.


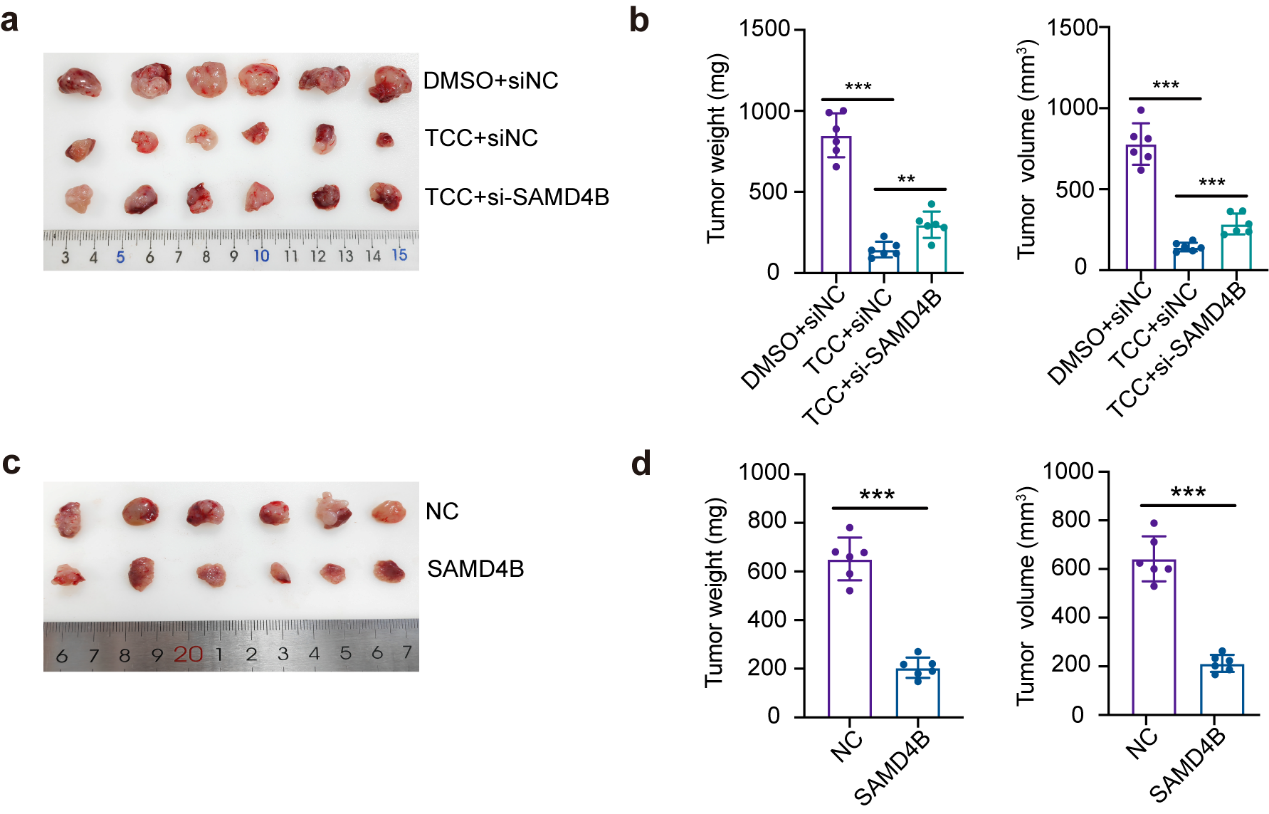


Figure S12. SAMD4B was activated by TCC therapy to achieve anti-tumor effect. (a) SAMD4B was knocked-down in the immunocompetent C57BL6/J mice, which were constructed orthotopic tumor and treated by TCC. (b) The expression of SAMD4B reduced to weaken the anti-tumor effect. (c) SAMD4B was overexpressed in the immunocompetent C57BL6/J mice, which were constructed orthotopic tumor and treated by TCC. (d) The expression of SAMD4B increased to improve the anti-tumor effect. Student’s test. (Level of significance: ***, *P* < 0.001; **, *P* < 0.01).


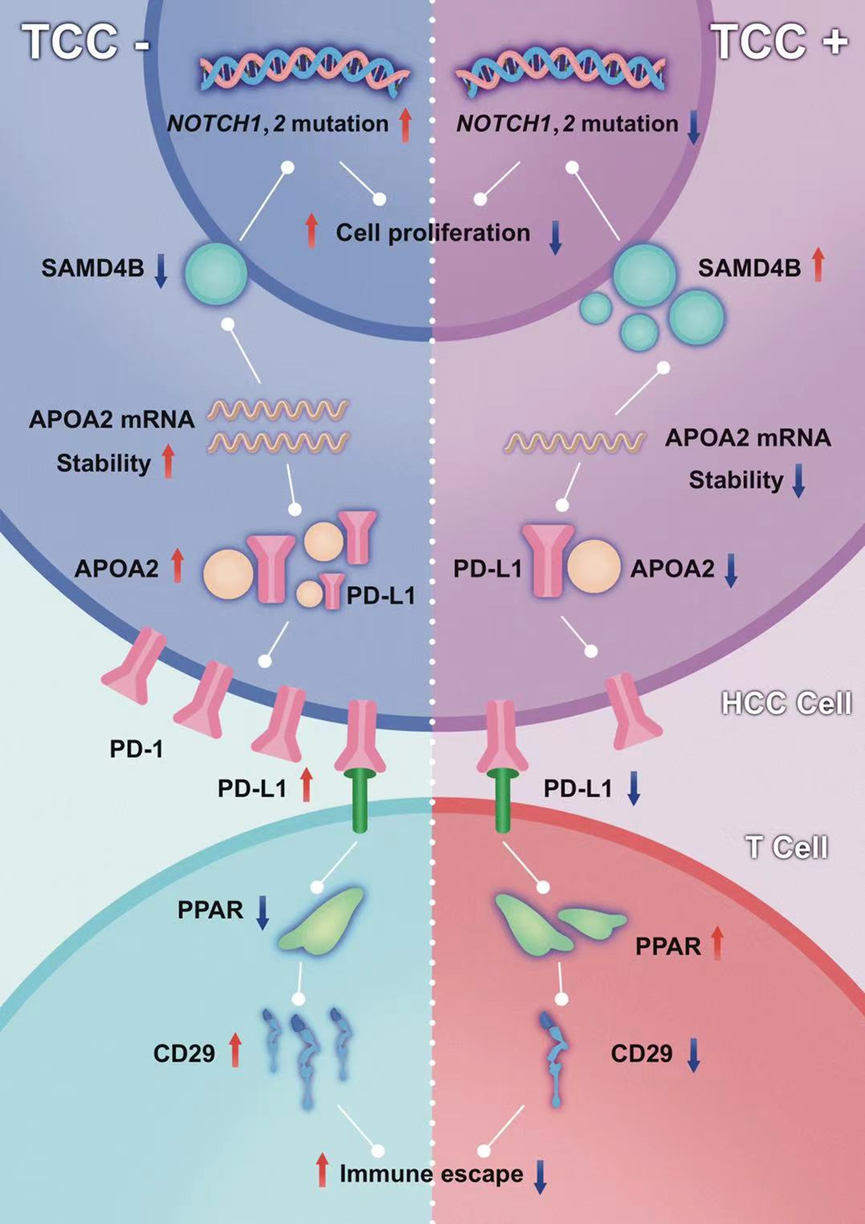


Figure S13. The mechanism of synergistic immunochemotherapy. Synergistic immunochemotherapy exerts an efficient anti-HCC effect by inducing the SAMD4B-APOA2 axis to inhibit tumour immune evasion.
