# Supplementary material for: Synergistic immunochemotherapy targeted SAMD4B-APOA2-PD-L1 axis potentiates antitumor immunity in hepatocellular carcinoma
Source: Cell Death Dis. 2024 Jun 17;15(6):421. doi: 10.1038/s41419-024-06699-2 (PMC11183041; doi:10.1038/s41419-024-06699-2)
Supplement: Supplementary file 6 — original data [file 41419_2024_6699_MOESM6_ESM.zip › orignal data/Original Western Blots.pdf]

Figure 5f

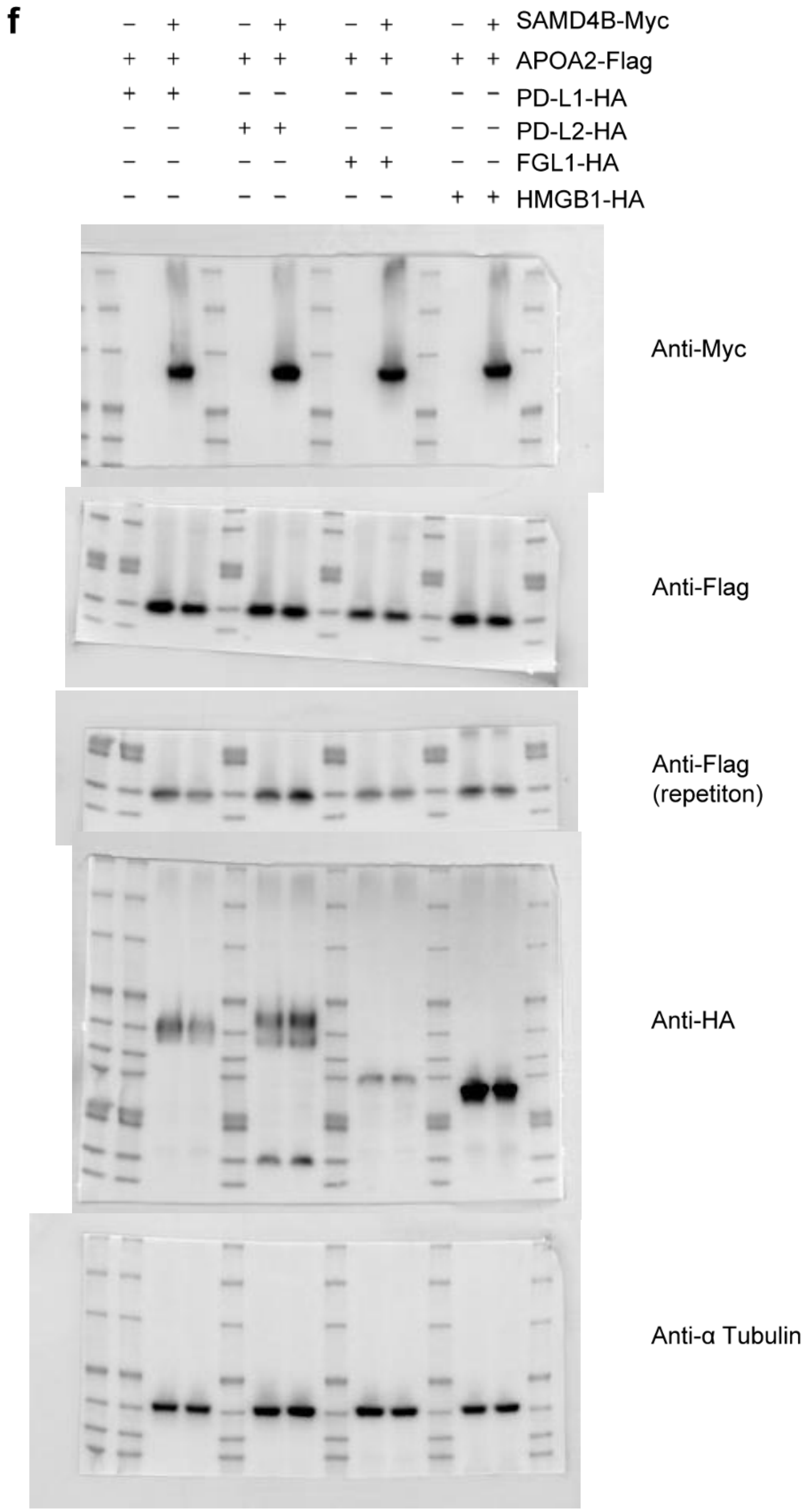
f
− + − + − + − + SAMD4B-Myc
+ + + + + + + + APOA2-Flag
+ + − − − − − − PD-L1-HA
− − + + − − − − PD-L2-HA
− − − − + + − − FGL1-HA
− − − − − − + + HMGB1-HA
Anti-Myc
Anti-Flag
Anti-Flag (repetiton)
Anti-HA
Anti-α Tubulin

Figure 5g

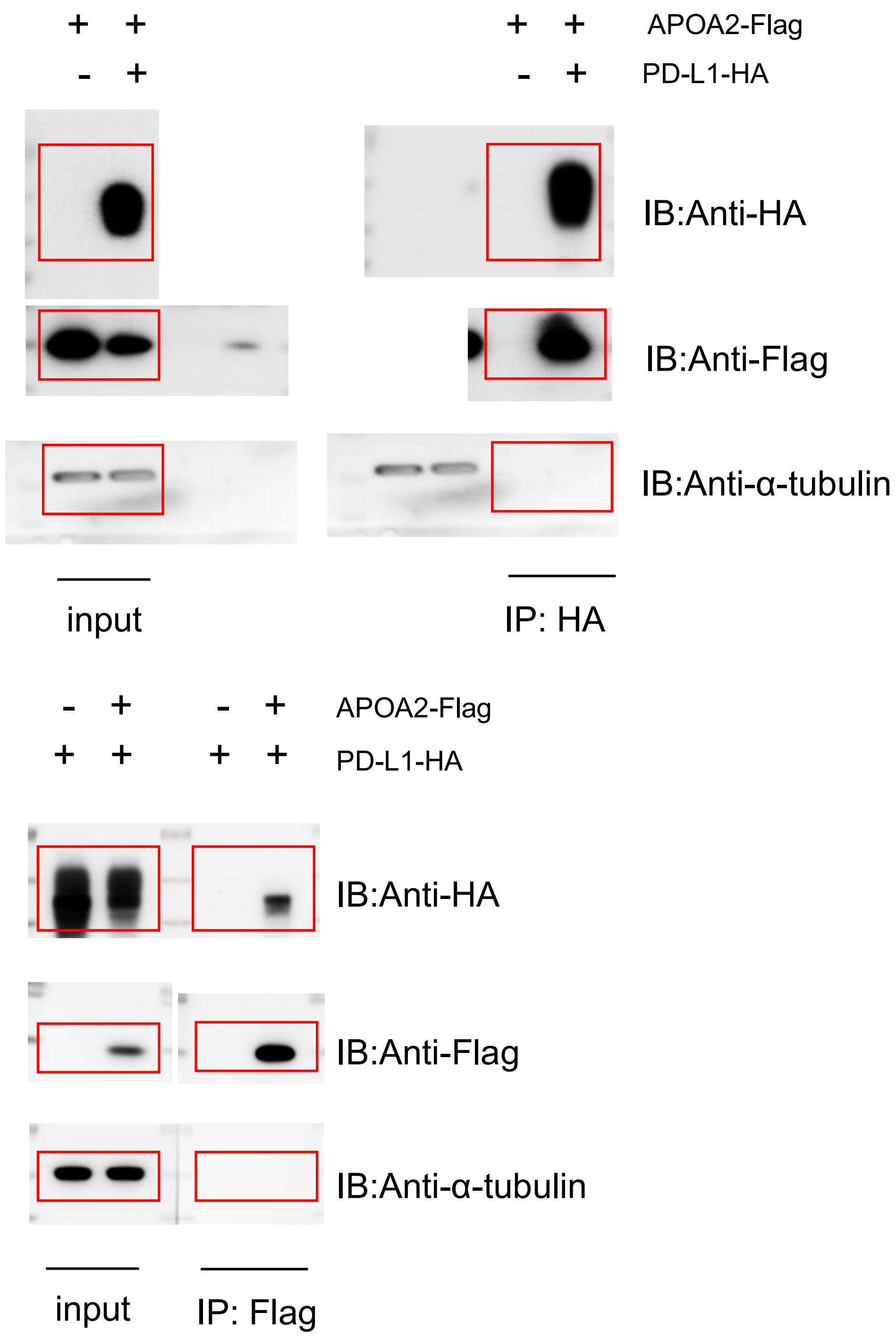
+ +
- +
+ +
- +
APOA2-Flag
PD-L1-HA
IB:Anti-HA
IB:Anti-Flag
IB:Anti-α-tubulin
input
IP: HA
- +
+ +
- +
+ +
APOA2-Flag
PD-L1-HA
IB:Anti-HA
IB:Anti-Flag
IB:Anti-α-tubulin
input
IP: Flag
