# Supplementary figures and images for: Synergistic immunochemotherapy targeted SAMD4B-APOA2-PD-L1 axis potentiates antitumor immunity in hepatocellular carcinoma
Source: Cell Death Dis. 2024 Jun 17;15(6):421. doi: 10.1038/s41419-024-06699-2 (PMC11183041; doi:10.1038/s41419-024-06699-2)

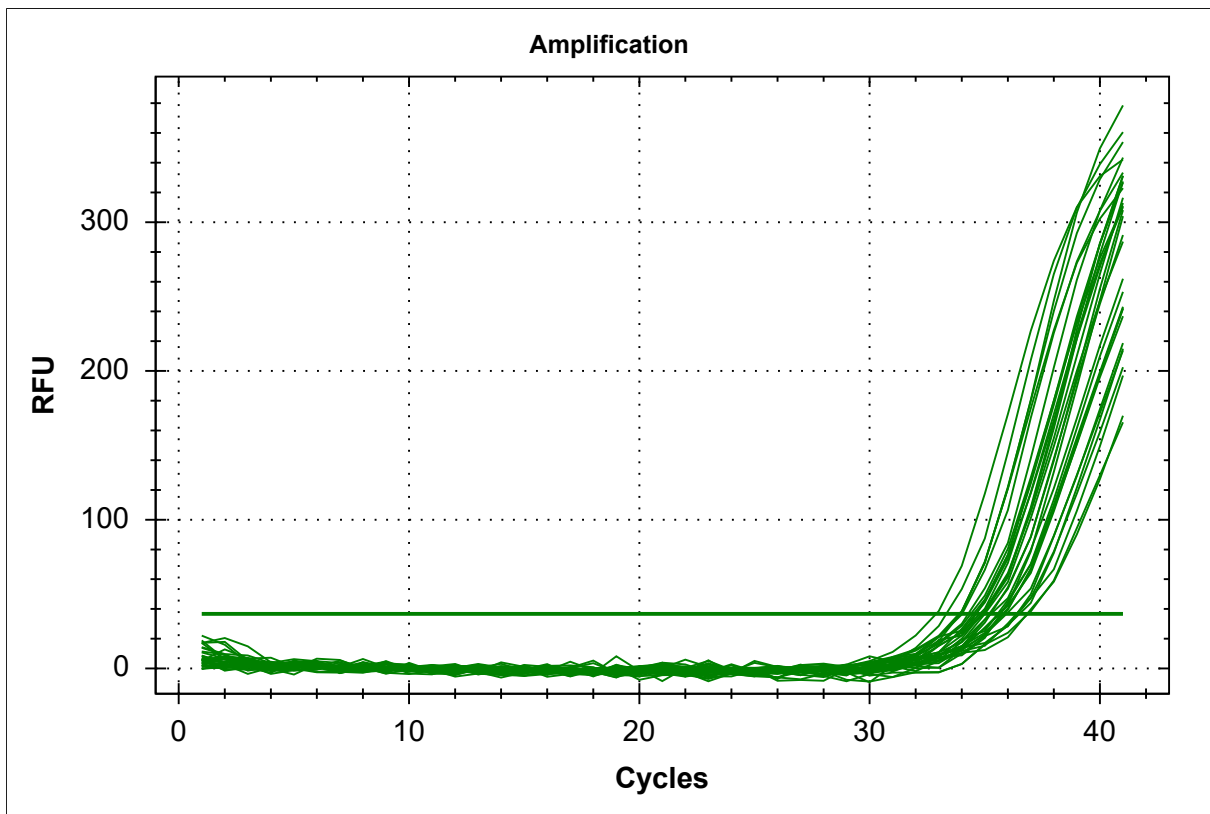

Supplement: Supplementary file 6 — original data [file 41419_2024_6699_MOESM6_ESM.zip › orignal data/qPCR/OE └⌐╘÷╟·╧▀.pdf]

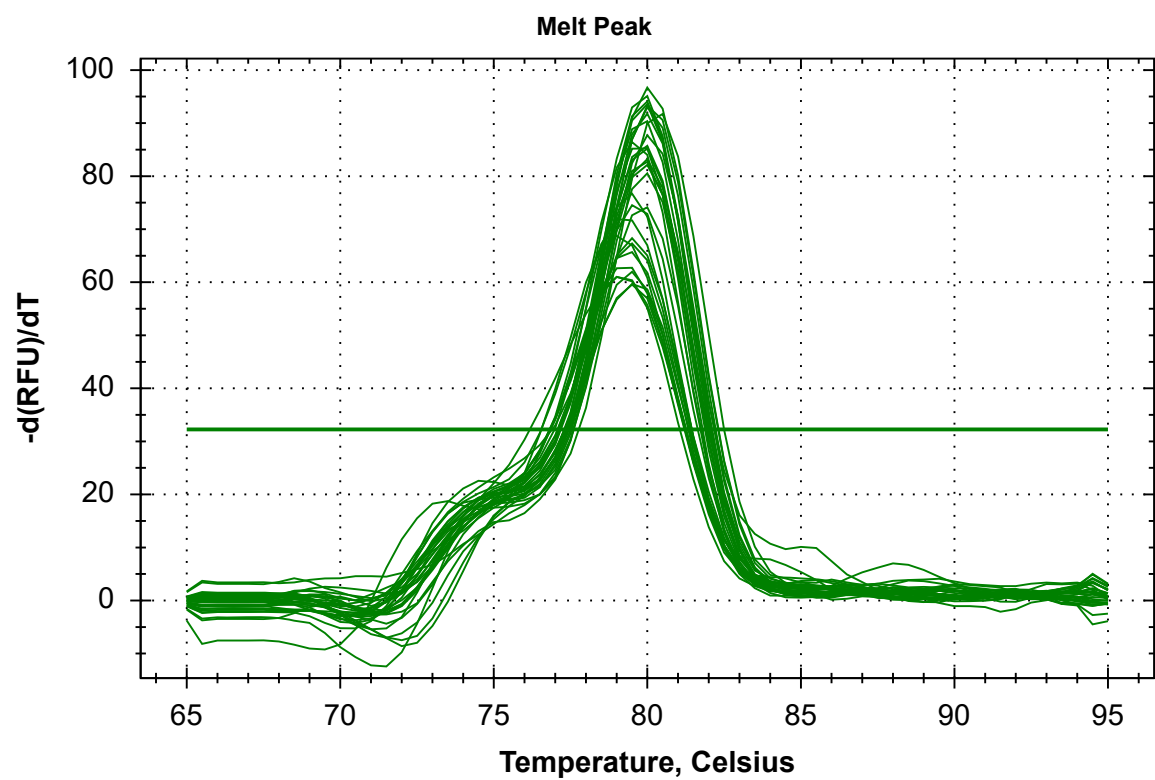

Supplement: Supplementary file 6 — original data [file 41419_2024_6699_MOESM6_ESM.zip › orignal data/qPCR/OE ╚▄╜Γ╖σ.pdf]

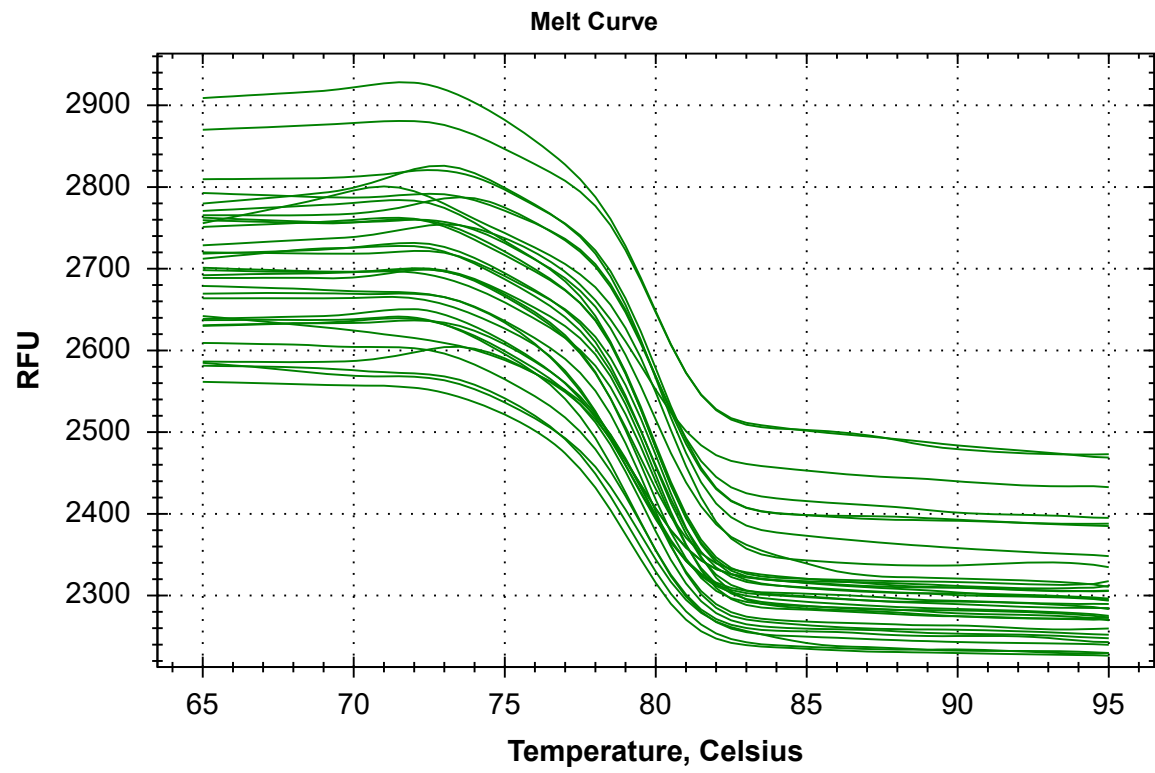

Supplement: Supplementary file 6 — original data [file 41419_2024_6699_MOESM6_ESM.zip › orignal data/qPCR/OE ╚▄╜Γ╟·╧▀.pdf]

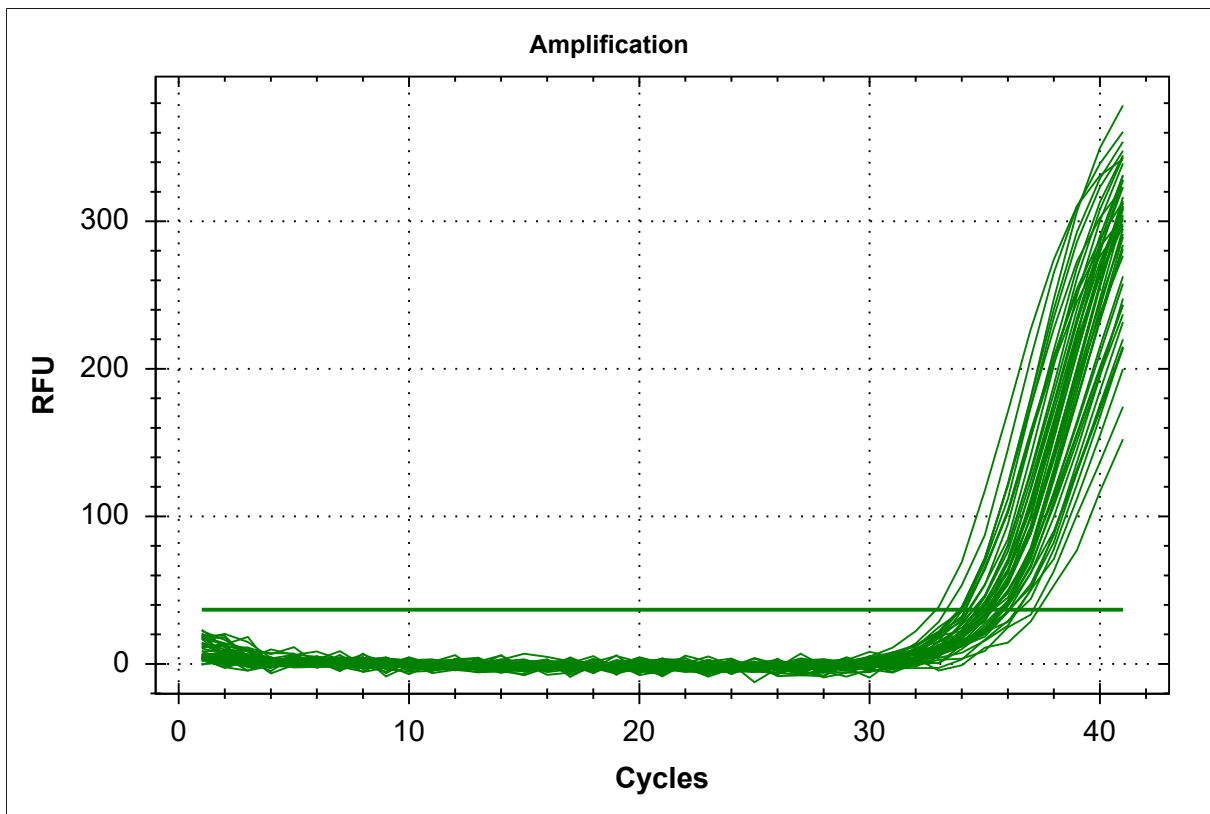

Supplement: Supplementary file 6 — original data [file 41419_2024_6699_MOESM6_ESM.zip › orignal data/qPCR/si └⌐╘÷╟·╧▀.pdf]

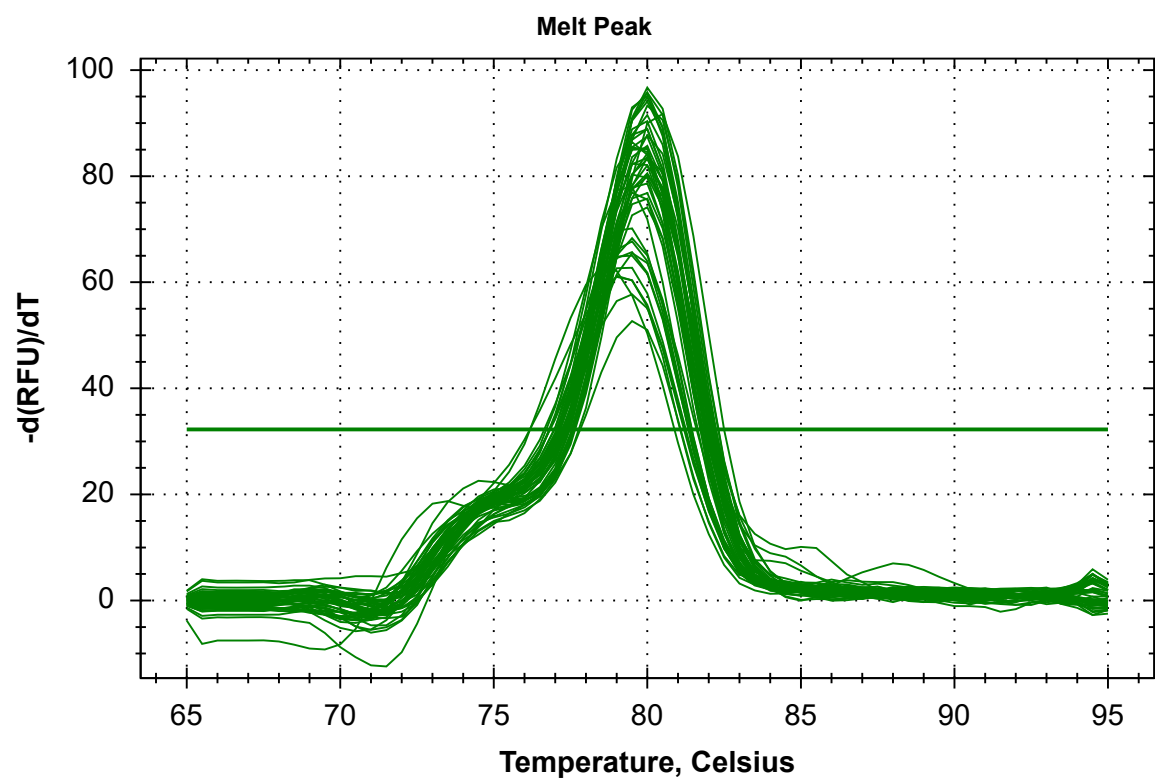

Supplement: Supplementary file 6 — original data [file 41419_2024_6699_MOESM6_ESM.zip › orignal data/qPCR/si ╚▄╜Γ╖σ.pdf]

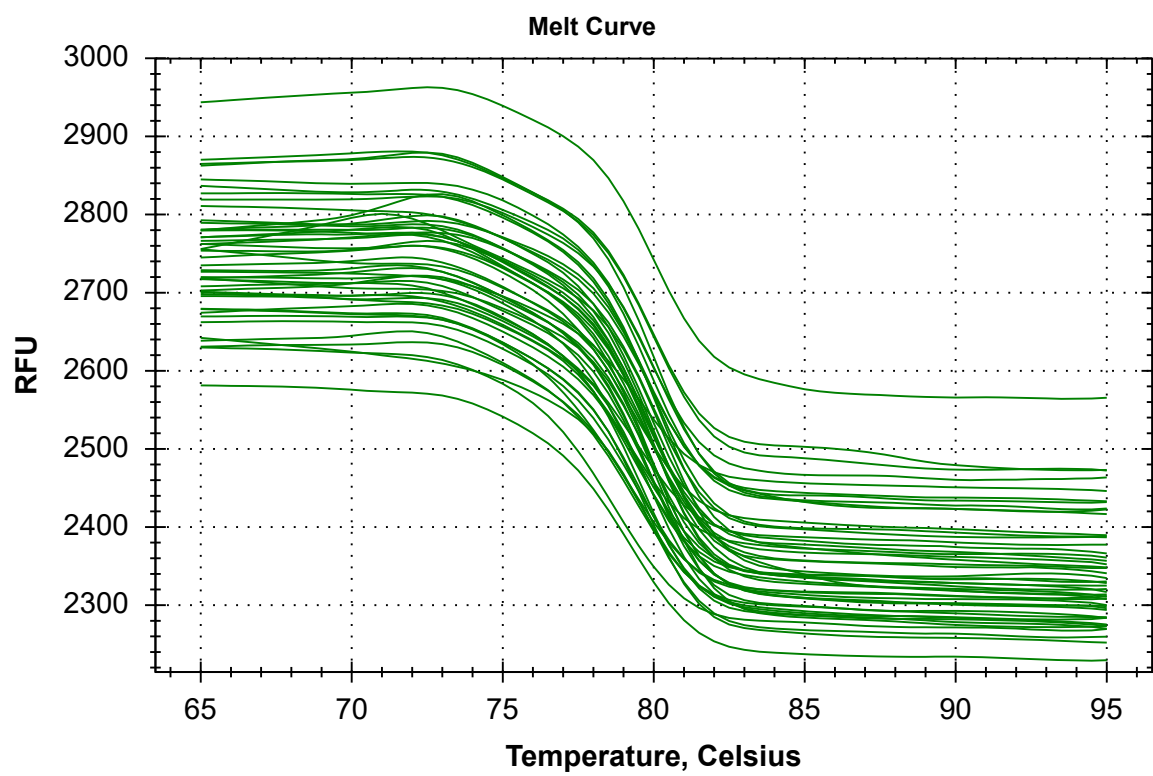

Supplement: Supplementary file 6 — original data [file 41419_2024_6699_MOESM6_ESM.zip › orignal data/qPCR/si ╚▄╜Γ╟·╧▀.pdf]
